# Landscape of copy number variants in Spanish people with dementia

**DOI:** 10.1038/s41525-026-00589-6

**Published:** 2026-06-30

**Authors:** Itziar de Rojas, Pablo García-González, Clàudia Olivé, Raquel Puerta, Laura Montrreal, Montserrat Alegret, Oscar Sotolongo-Grau, Amanda Cano, Marta Marquié, Sergi Valero, Miguel Calero, Alberto Rábano, Ana Belén Pastor, Teodoro del Ser, Inés Quintela, Juan Macías, Anaïs Corma-Gómez, Juan A. Pineda, Emilio Franco-Macías, Dolores Buiza-Rueda, María Bernal Sánchez-Arjona, Jose Luis Royo, Silvia Mendoza, Carmen Lage, Carmen Antúnez, Arturo Corbatón-Anchuelo, María Teresa Martínez-Larrad, Mónica Diez-Fairen, Ignacio Alvarez, Raquel Huerto Vilas, Alfonso Arias Pastor, Manuel Menéndez-González, Carmen Martínez Rodríguez, Irene Rosas Allende, Sebastián García-Madrona, Ana Frank-García, Angel Martín-Montes, Miquel Baquero, GR@ACE, Oscar Sotolongo-Grau, Juan A. Pineda, Nuria Aguilera, Montserrat Alegret, Paula Bayón-Buján, Natali Bein, Josep Blazquez-Folch, Mercè Boada, Mar Buendia, Amanda Cano, Berta Calm, Pilar Cañabate, Maria Capdevila-Bayo, Angel Carracedo, A. Corbatón-Anchuelo, Federico Casales, Itziar de Rojas, Susana Diego, Ana Espinosa, Maria Victoria Fernandez, Anna Gailhajenet, Pablo García-González, Marina Guitart, Marta Ibarria, Asunción Lafuente, Núria Lleonart, Juan Macias, Olalla Maroñas, Elvira Martín, Maria Teresa Martínez, Marta Marquié, Andrea Miguel, Laura Montrreal, Xavier Morató, Mariola Moreno, Nathalia Muñoz, Álvaro Muñoz-Morales, Clàudia Olivé, Gemma Ortega, Ana Pancho, Ester Pelejà, Alba Pérez-Cordon, Raquel Puerta, Silvia Preckler, Inés Quintela, Luis Miguel Real, José Nelet Rodríguez, Maitee Rosende-Roca, Agustín Ruiz, Maria Eugenia Sáez, Angela Sanabria, Susanna Seguer, Luís Tárraga, Juan Pablo Tartari, Sergi Valero, Liliana Vargas, DEGESCO, Nuria Aguilera, Paula Bayón-Buján, Mar Buendia, Amanda Cano, Susana Diego, Anna Gailhajenet, Pablo García-González, Marina Guitart, Marta Ibarria, Asunción Lafuente, Olalla Maroñas, Elvira Martín, Laura Montrreal, Mariola Moreno, Clàudia Olivé, Ana Pancho, Raquel Puerta, Silvia Preckler, Liliana Vargas, Astrid Daniela Adarmes-Gómez, Miquel Aguilar, Daniel Alcolea, Montserrat Alegret, María Dolores Alonso, Ignacio Alvarez, Victoria Álvarez, Guillermo Amer-Ferrer, Martirio Antequera, Anna Antonell, Carmen Antúnez, Alfonso Arias Pastor, Miquel Baquero, Olivia Belbin, María Bernal Sánchez-Arjona, Mercè Boada, Marta Bonilla-Toribio, Dolores Buiza-Rueda, María Jesús Bullido, Maria Teresa Buongiorno, Miguel Calero, Ana Camara, Pilar Cañabate, Fernando Cardona Serrate, Ángel Carracedo, Fátima Carrillo, María José Casajeros, Consuselo Chafer Pericás, Yaroslau Compta, Arturo Corbatón-Anchuelo, Anaïs Corma-Gómez, Paz de la Guía, Itziar de Rojas, Teodoro del Ser, Rafael Díaz Belloso, Mónica Diez-Fairen, Oriol Dols-Icardo, María Elena Erro Aguirre, Ana Espinosa, Manuel Fernandez, Maria Victoria Fernández, Marta Fernández-Fuertes, Juan Fortea, Emilio Franco-Macías, Ana Frank-García, Jose María García-Alberca, Sergio García-Díaz, Sebastián García-Madrona, Guillermo Garcia-Ribas, Ernesto García-Roldán, Lorena Garrote-Espina, Pilar Gómez-Garre, Raquel Huerto Vilas, Ignacio Illán-Gala, Silvia Jesús, Jaime Kulisevsky, Miguel Angel Labrador Espinosa, Carmen Lage, Agustina Legaz, Alberto Lleó, Sara López-García, Adolfo Lopez de Munain, Juan Macías, Daniel Macias-García, Salvadora Manzanares, Marta Marín, Juan Marín-Muñoz, Marta Marquié, Maria Marti, Miguel Martín-Bórnez, Angel Martín-Montes, Angel Martín Montés, Victoriana Martínez, Begoña Martínez, Pablo Martínez-Lage Álvarez, María Teresa Martínez-Larrad, Marian Martinez de Pancorbo, Carmen Martínez Rodríguez, Miguel Medina, Maite Mendioroz Iriarte, Silvia Mendoza, Manuel Menéndez-González, Pablo Mir, Laura Molina-Porcel, Fermin Moreno, Esteban Muñoz, Laura Muñoz-Delgado, Fuensanta Noguera Perea, Gemma Ortega, Javier Pagonabarraga, Celia Painous, Ana Belén Pastor, Pau Pastor, Ester Pelejá, Alba Pérez-Cordón, Jordi Pérez-Tur, María Teresa Periñán, Juan Antonio Pineda, Rocío Pineda-Sánchez, Gerard Piñol-Ripoll, Inés Quintela, Alberto Rábano, Luis Miguel Real, Diego Real de Asúa, Eloy Rodriguez-Rodriguez, Irene Rosas Allende, Maitée Rosende-Roca, Jose Luis Royo, Agustín Ruiz, María Eugenia Sáez, Ángela Sanabria, Pascual Sánchez-Juan, Raquel Sánchez-Valle, Javier Sánchez-Ruiz de Gordoa, Isabel Sastre, Oscar Sotolongo-Grau, Lluís Tárraga, Sergi Valero, Francesc Valldeoriola, María Pilar Vicente, Laura Vivancos-Moreau, Jordi Pérez-Tur, María J. Bullido, Guillermo Garcia-Ribas, Victoria Álvarez, Gerard Piñol-Ripoll, Pau Pastor, Eloy Rodriguez-Rodriguez, Jose María García-Alberca, Pablo Mir, Luis M. Real, Miguel Medina, María Eugenia Sáez, Ángel Carracedo, Michael T. Heneka, Lluís Tàrraga, Mercè Boada, Pascual Sánchez-Juan, M. Victoria Fernández, Sven J. van der Lee, Agustín Ruiz

**Affiliations:** 1 Ace Alzheimer Center Barcelona, Universitat Internacional de Catalunya, Barcelona, Spain; 2 CIBERNED, Network Center for Biomedical Research in Neurodegenerative Diseases, National Institute of Health Carlos III, Madrid, Spain; 3 Luxembourg Centre for Systems Biomedicine (LCSB), University of Luxembourg, Esch-sur-Alzette, Luxembourg; 4 Doctorate in Biotecnology, Faculty of Pharmacy and Food Sciences, University of Barcelona, Barcelona, Spain; 5 CIEN Foundation/Queen Sofia Foundation Alzheimer Center/Instituto de Salud Carlos III, Madrid, Spain; 6 UFIEC, Instituto de Salud Carlos III, Madrid, Spain; 7 CIEN Foundation/Queen Sofia Foundation Alzheimer Center, Madrid, Spain; 8 BT-CIEN, Madrid, Spain; 9 Department of Neurology/CIEN Foundation/Queen Sofia Foundation Alzheimer Center, Madrid, Spain; 10 Grupo de Medicina Xenómica, Fundación Pública Galega de Medicina Xenómica, Santiago de Compostela, Spain; 11 Instituto de Biomedicina de Sevilla (IBIS)/Departamento de Medicina, Universidad de Sevilla, Sevilla, Spain; 12 CIBERINFEC, Network Center for Biomedical Research in Infectious Diseases, National Institute of Health Carlos III, Madrid, Spain; 13 Unidad Clínica de Enfermedades Infecciosas y Microbiología. Hospital Universitario de Valme, Sevilla, Spain; 14 Unidad de Demencias, Servicio de Neurología y Neurofisiología. Instituto de Biomedicina de Sevilla (IBiS), Hospital Universitario Virgen del Rocío/CSIC/Universidad de Sevilla, Sevilla, Spain; 15 Unidad de Trastornos del Movimiento, Servicio de Neurología y Neurofisiología. Instituto de Biomedicina de Sevilla (IBiS), Hospital Universitario Virgen del Rocío/CSIC/Universidad de Sevilla, Sevilla, Spain; 16 Departamento de Especialidades Quirúrgicas, Bioquímica e Inmunología. School of Medicine. University of Malaga, Málaga, Spain; 17 Alzheimer Research Center & Memory Clinic, Instituto Andaluz de Neurociencia, Málaga, Spain; 18 Neurology Service, Marqués de Valdecilla University Hospital (University of Cantabria and IDIVAL), Santander, Spain; 19 Unidad de Demencias, Hospital Clínico Universitario Virgen de la Arrixaca, Murcia, Spain; 20 Instituto de Investigación Sanitaria, Hospital Clínico San Carlos (IdISSC), Madrid, Spain; 21 Spanish Biomedical Research Centre in Diabetes and Associated Metabolic Disorders (CIBERDEM), Madrid, Spain; 22 Fundació Docència i Recerca MútuaTerrassa, Terrassa, Spain; 23 Memory Disorders Unit, Department of Neurology, Hospital Universitari Mutua de Terrassa, Terrassa, Barcelona, Spain; 24 Unitat Trastorns Cognitius, Hospital Universitari Santa Maria de Lleida, Lleida, Spain; 25 Institut de Recerca Biomedica de Lleida (IRBLLeida), Lleida, Spain; 26 Servicio de Neurología. Hospital Universitario Central de Asturias, Oviedo, Spain; 27 Instituto de Investigación Sanitaria del Principado de Asturias (ISPA), Asturias, Spain; 28 Departamento de Medicina, Universidad de Oviedo, Oviedo, Spain; 29 Hospital de Cabueñes, Gijón, Spain; 30 Laboratorio de Genética. Hospital Universitario Central de Asturias, Oviedo, Spain; 31 Hospital Universitario Ramon y Cajal, IRYCIS, Madrid, Spain; 32 Department of Neurology, La Paz University Hospital. Instituto de Investigación Sanitaria del Hospital Universitario La Paz. IdiPAZ, Madrid, Spain; 33 Hospital La Paz Institute for Health Research, IdiPAZ, Madrid, Spain; 34 Universidad Autónoma de Madrid, Madrid, Spain; 35 Department of Neurology, La Paz University Hospital, Madrid, Spain; 36 Servei de Neurologia, Hospital Universitari i Politècnic La Fe, Valencia, Spain; 37 Grupo de Investigación en Enfermedad de Alzheimer, GINEA, Instituto de Investigación Sanitaria La Fe, Valencia, Spain; 38 Unitat de Genètica Molecular, Institut de Biomedicina de València-CSIC, Valencia, Spain; 39 Centro de Biología Molecular Severo Ochoa (UAM-CSIC), Madrid, Spain; 40 Instituto de Investigacion Sanitaria ‘Hospital la Paz’ (IdIPaz), Madrid, Spain; 41 Unit of Neurodegenerative diseases, Department of Neurology, Hospital Germans Trias i Pujol, Badalona, Barcelona, Spain; 42 Neurodegenerative Diseases Research Laboratory, Germans Trias i Pujol Research Laboratory, Badalona, Barcelona, Spain; 43 Unidad de Trastornos del Movimiento, Servicio de Neurología, Instituto de Biomedicina de Sevilla, Hospital Universitario Virgen del Rocío/CSIC/Universidad de Sevilla, Sevilla, Spain; 44 Departamento de Medicina, Facultad de Medicina, Universidad de Sevilla, Sevilla, Spain; 45 Department of Medical Biochemistry, Molecular Biology and Immunology, University of Sevilla, Sevilla, Spain; 46 CAEBI, Centro Andaluz de Estudios Bioinformáticos, Sevilla, Spain; 47 Grupo de Medicina Xenómica, CIBERER, CIMUS. Universidade de Santiago de Compostela, Santiago de Compostela, Spain; 48 Fundación Pública Galega de Medicina Xenómica- IDIS, Santiago de Compostela, Spain; 49 Alzheimer’s Centre Reina Sofia-CIEN Foundation-ISCIII, Madrid, Spain; 50 Genomics of Neurodegenerative Diseases and Aging, Human Genetics, Vrije Universiteit Amsterdam, Amsterdam UMC location VUmc, Amsterdam, The Netherlands; 51 Alzheimer Center Amsterdam, Neurology, Vrije Universiteit Amsterdam, Amsterdam UMC location VUmc, Amsterdam, The Netherlands; 52 Amsterdam Neuroscience, Neurodegeneration, Amsterdam, The Netherlands; 53 Biggs Institute for Alzheimer’s and Neurodegenerative Diseases, University of Texas Health Science Center, San Antonio, TX USA; 54 Networking Research Center on Neurodegenerative Diseases (CIBERNED), Instituto de Salud Carlos III, Madrid, Spain; 55 Grupo de Medicina Xenómica, Centro Nacional de Genotipado (CEGEN-PRB3-ISCIII). Universidad de Santiago de Compostela, Santiago de Compostela, Spain; 56 Fundación Pública Galega de Medicina Xenómica - CIBERER-IDIS, Santiago de Compostela, Spain; 57 Centro de Investigación Biomédica en Red de Diabetes y Enfermedades Metabólicas Asociadas, CIBERDEM, Madrid, Spain; 58 Hospital Clínico San Carlos, Madrid, Spain; 59 Department of Neurology, II B Sant Pau, Hospital de la Santa Creu i Sant Pau, Universitat Autònoma de Barcelona, Barcelona, Spain; 60 Servei de Neurologia. Hospital Clínic Universitari de València, Valencia, Spain; 61 Department of Neurology, Hospital Universitario Son Espases, Palma, Spain; 62 Unidad de Demencias, Hospital Clínico Universitario Virgen de la Arrixaca, Palma, Spain; 63 Alzheimer’s disease and other cognitive disorders unit. Service of Neurology. Hospital Clínic of Barcelona. Institut d’Investigacions Biomèdiques August Pi i Sunyer, University of Barcelona, Barcelona, Spain; 64 Parkinson’s Disease & Movement Disorders Unit, Neurology Service, Hospital Cliínic i Universitari de Barcelona, Barcelona, Spain; 65 IDIBAPS, CIBERNED, ERN-RND, Institut Cliínic de Neurociències UBNeuro (Maria de Maeztu Excellence Centre), Universitat de Barcelona, Barcelona, Spain; 66 Unidad Mixta de Neurologia Genètica, Instituto de Investigación Sanitaria La Fe, Valencia, Spain; 67 Navarrabiomed, Pamplona, Spain; 68 Department of Neurology, Hospital Universitario de Navarra, Pamplona, Spain; 69 Instituto de Investigación Sanitaria de Navarra (IDISNA), Pamplona, Spain; 70 Universitat de Barcelona (UB), Barcelona, Spain; 71 Sant Pau Memory Unit, Department of Neurology, Institut de Recerca de Sant Pau; Hospital de Sant Pau, Barcelona, Spain; 72 Movement Disorders Unit, Department of Neurology, Institut de Recerca de Sant Pau; Hospital de Sant Pau, Barcelona, Spain; 73 Service of Neurology, University Hospital Marqués de Valdecilla, IDIVAL, University of Cantabria, Santander, Spain; 74 Department of Neurology. Hospital Universitario Donostia, San Sebastian, Spain; 75 Department of Neurosciences. Faculty of Medicine and Nursery. University of the Basque Country, San Sebastián, Spain; 76 Neurosciences Area. Instituto Biodonostia, San Sebastian, Spain; 77 Centro de Investigación y Terapias Avanzadas. Fundación CITA-alzheimer, San Sebastian, Spain; 78 BIOMICS País Vasco; Centro de investigación Lascaray, Universidad del País Vasco UPV/EHU, Vitoria-Gasteiz, Spain; 79 Neurological Tissue Bank of the Biobanc-Hospital Clinic-IDIBAPS, Institut d’Investigacions Biomèdiques August Pi i Sunyer, Barcelona, Spain; 80 Centre for Preventive Neurology, Wolfson Institute of Population Health, Queen Mary University of London, London, UK; 81 Hospital Universitario La Princesa, Madrid, Spain

**Keywords:** Diseases, Genetics, Neurology, Neuroscience

## Abstract

Recent studies suggest that copy number variants (CNVs) may contribute to the missing heritability of complex diseases such as Alzheimer’s disease (AD) and related dementias (ADRD). We performed a CNV analysis using genotyping data (Axiom 815 K Spanish biobank array) from the GR@ACE/DEGESCO dementia dataset (*n* = 20,067) of the Spanish population. Applying PennCNV and extensive quality control, 8275 controls and 7818 dementia cases were selected for gene-level case/control associations. We identified 43,833 CNVs with deletions (47%) and duplications (53%). No genome-wide significant associations were found, but nominal associations were observed in *PKP3*-*SIGIRR* and *FBRSL1* loci. CNVs in 2970 genes were exclusive to dementia cases and enriched in vascular-related pathways. Notable findings included 14q11.2 duplication and *VPS13B* deletions in ADRD cases, the latter confirmed by optical genome mapping. Our findings suggest potential novel genes associated with ADRD in the Spanish population. However, the limited resolution of array-based technologies in detecting CNVs warrants further investigation.

## Introduction

Dementia is a clinical syndrome resulting from a variety of underlying conditions—both neurodegenerative and non-neurodegenerative—characterized by progressive cognitive decline and loss of functional independence. It poses a major global health challenge, affecting millions of people worldwide^1^. Genetics plays a critical role in the risk landscape of Alzheimer’s disease (AD) and related dementias (ADRD), as heritability estimates suggest that ~70–80% of disease susceptibility can be attributed to genetic factors^2^. Consequently, numerous genome-wide association studies (GWAS) and sequencing efforts have identified common and rare variants associated with these ADRD, substantially advancing our understanding of their genetic architecture^3–5^. However, despite these advances, a significant proportion of heritability remains unexplained, suggesting additional genomic mechanisms at play. Among these, copy number variations (CNVs) remain relatively underexplored in ADRD^6,7^, leaving important gaps in our understanding of their role in neurodegeneration.

CNVs are one of the most prevalent types of structural variations (SV) in the human genome, involving gain (duplication) or loss (deletion) of specific segments of DNA that vary from one kilobase (kb) to several megabases (Mb)^8^. Due to their large size, many CNVs span or disrupt multiple genes, resulting in altered gene dosage and impaired biological function^9^. This mechanism can underlie not only isolated gene disorders but also the development of contiguous gene syndromes or CNV syndromes (CGSs) caused by deletions or duplications that affect several neighboring genes^10^. Some CGSs, such as 22q11.2 deletion syndrome, 1q21.1 and 16p11.2 CNV syndromes, and Williams–Beuren syndrome (7q11.23 deletion), present with cognitive impairment, developmental delay, or even progressive neuropsychiatric symptoms^11^ that overlap with dementia phenotypes.

Importantly, several CNVs have been linked to several complex disorders, including neurodegenerative diseases^9^ such as amyotrophic lateral sclerosis (e.g., *C9orf72* repeat expansions)^12,13^, Parkinson’s disease (*SNCA* duplications)^14,15^ and AD (*APP* locus duplications)^8,16–20^. Beyond neurodegeneration, CNVs also increase risk for psychiatric and neurodevelopmental conditions such as schizophrenia^21^, autism, and intellectual disability^22,23^. These genomic rearrangements can alter gene expression, disrupt critical biological pathways involved in neuronal function, synaptic plasticity, immune response, and protein homeostasis, ultimately contributing to the onset and progression of dementia symptoms.

Recent advances in genomic technologies now allow systematic investigation of CNVs across large cohorts, revealing that up to 12% of the human genome and thousands of genes are variable in copy number, responsible for an important proportion of normal phenotypic variation^24,25^. However, CNV detection in next-generation sequencing (NGS) data requires high-sequencing depth and uniformity of coverage, which may not be achievable in a cost- and time-effective manner^26,27^. Consequently, microarray-based CNV detection and its calling algorithms continue to be commonly used clinical genetic test^28^, offering an optimal compromise between cost and resolution and enabling the reuse of extensive existing datasets for research.

Current challenges include linking CNVs to biological function and understanding their role in common and complex human diseases. Large-scale GWAS and integrative multi-omics approaches are now essential to identify novel CNVs associated with dementia and decipher their functional consequences^29,30^. Addressing these gaps could advance personalized medicine strategies and guide the development of targeted therapies.

Here, we investigated the genome-wide landscape of CNVs and their associations with ADRD in a large cohort of 20,067 participants from the Spanish GR@ACE/DEGESCO consortium in order to further characterize the genetic causes of dementia in this population. We report CNVs overlapping known genes such as *APP, MAPT* and *ABCA7*. Additionally, we also describe candidate novel CNVs potentially associated with dementia, overlapping the genes *FBRSL1, PKP3-SIGIRR, VPS13B* and 14q11.2 region.

## Results

### Sample demographics and CNV call characteristics

We analyzed a total of 43,833 CNVs across 16,093 individuals, including 8275 controls and 7818 dementia cases. Dementia cases included a significantly higher proportion of females (68.6% vs. 50.0% in controls) and were substantially older, as expected (mean age: 79.9 vs. 56.9 years), compared to the population control group. However, the proportion of individuals carrying at least one CNV was comparable between groups (88.7% in controls vs. 88.0% in dementia, *p* = 0.208, Table 1). Overall, the detected CNV represented 19,579 unique genomic regions. Notably, only 2380 (12.2%) CNV regions were shared between cases and controls, indicating that the majority of CNV regions were exclusive to either group, reflecting a high degree of non-overlap in CNV distribution. On average, control individuals carried more CNVs (2.81 vs 2.64, *p* = 3.2 × 10^−03^). As expected, CNV length and the number of SNPs within each CNV were strongly correlated overall (*r* = 0.82), supporting the reliability of probe density as a proxy for CNV size. Interestingly, this correlation was stronger among controls (*r* = 0.85) compared to dementia cases (*r* = 0.76). This may help explain why, although dementia cases having slightly longer CNVs on average (272.7 kb vs. 262.3 kb, *p* = 3.06 × 10^−02^), the average number of SNPs within CNVs was lower (71.0 vs. 78.9 SNPs, *p* = 3.4 × 10^−04^). In addition, regression analysis adjusted by sex and diagnosis showed that age was positively associated with CNV length (*p* = 1.2 × 10^−03^), while no significant association was observed with the number of SNPs per CNV (*p* = 0.21). These differences suggest that CNVs in dementia cases may more frequently affect genomic regions with lower SNP density, and that age-related increases in CNV size do not necessarily correspond to higher probe density. Nevertheless, the number of genes affected per CNV and per individual was largely comparable across groups (*p* > 0.05, Table 1).

**Table 1:** Cohort demographic and CNV characteristics

	Control	Dementia	Total	*P* value*
Individuals (*n*,%)	8275 (51.42)	7818 (48.58)	16,093	-
Indiv. with CNVs (*n*,%)	7339 (88.7)	6883 (88.0)	14,222 (88.4)	0.208
Females (*n*,%)	4139 (50)	5362 (68.6)	9501 (59.0)	<2.2 × 10^−16^
Age (mean, sd)	56.9 (17.9)	79.9 (7.88)	68.17 (18.06)	<2.2 × 10^−16^
CNVs (*n*,%)	23,217 (52.97%)	20,616 (47.03%)	43,833	-
Unique	9498 (48.5%)	7701 (39.3%)	19,579	-
Per individual	2.81	2.64	2.72	3.16 × 10^−03^
SNPs (mean, sd)	78.9 (436)	71.0 (347)	75.2 (397.0)	3.42 × 10^−04^
Length (mean kb, sd)	262.3 (1503.7)	272.7 (1250.5)	267.2 (1390.4)	3.06 × 10^−02^
Genes affected by CNV (mean, sd)	2.93 (5.1)	2.94 (4.3)	2.94 (4.7)	0.989
Genes affected by indiv (mean, sd)	8.79 (34.6)	8.12 (22.0)	8.46 (29.18)	0.079
Duplications (*n*,%)	11,963 (51.8%)	11,115 (48.2%)	23,078	-
Per individual	1.45	1.42	1.43	0.060
SNPs (mean, sd)	96.5 (537)	87.5 (442)	92.2 (493)	1.42 × 10^−09^
Length (mb) min-max mean-sd	0.050–146.10 0.340 (1.933)	0.050–133.62 0.355 (1.640)	0.050–146.10 0.347 (1.798)	0.299
Genes affected by CNV (mean, sd)	3.91 (7.3)	3.89 (10.5)	3.9 (9.0)	0.247
Genes affected by indiv (mean, sd)	5.93 (22.6)	5.68 (19.4)	5.81 (21.1)	0.155
Deletions (*n*,%)	11254 (54.2%)	9501 (45.8%)	20,755	-
Per individual	1.36	1.22	1.29	0.041
SNPs (mean, sd)	60.1 (294)	51.8 (181)	56.3 (248)	0.135
Length (mb) min–max mean-sd	0.050–74.722 0.180 (0.825)	0.050–29.353 0.176 (0.477)	0.50–74.722 0.178 (0.688)	0.758
Genes affected by CNV (mean, sd)	2.20 (3.9)	2.25 (3.3)	2.2 (3.6)	0.976
Genes affected by indiv (mean, sd)	3.62 (19.0)	3.10 (12.4)	3.37 (16.1)	0.143

*Chi-square test used for comparisons of sex and proportion of individuals with CNVs; Wilcoxon rank-sum test used for all other comparisons.

Comparing duplications vs. deletions, duplications were significantly more frequent than deletions (23,078 vs. 20,755 events, *p* < 2.2 × 10^−16^); were consistently larger, contained more SNPs, and affected more genes per CNV and per individual (mean CNVrate_del-dup_ = 1.29–1.43, mean length_del-dup_ = 178–347.4 kb and mean genes_del-dup_ = 3.4–5.8; *p* < 2.2 × 10^−16^). When stratified by CNV type, duplications were slightly more frequent in controls (51.8%) than in dementia. On average, individuals carried ~1.4 duplications. Duplication length was comparable ( ~ 0.35 Mb), and they affected ~3.9 genes per CNV and ~5.8 genes per individual. Although gene burden did not differ significantly, duplications in individuals with dementia contained significantly fewer SNPs (*p* = 1.42 × 10^−09^). Deletions occurred significantly more frequently in controls (1.36 vs. 1.22; *p* = 0.041), though deletion length and gene content did not differ between groups (Table 1). Finally, linear regression models adjusted for age, sex and diagnosis showed no significant association between age or sex and the number of deletions or duplications per individual (*p* > 0.05).

### Case/control association analyses

#### Genome-wide approach

**Fig. 1:**
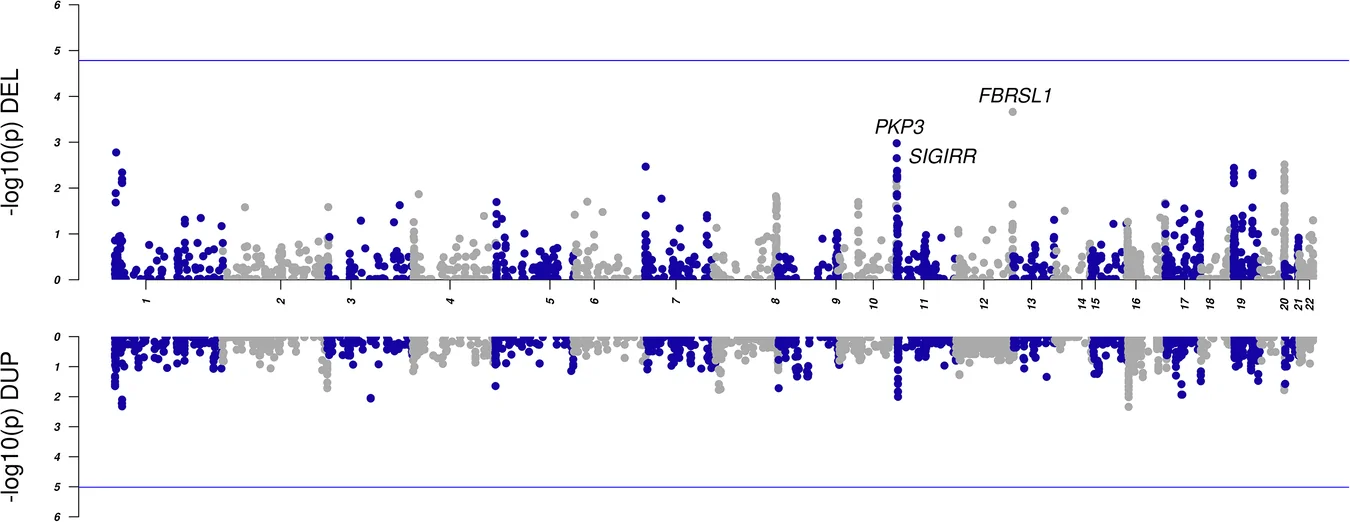
Miami plot for logistic regression in deletions (*λ* = 0.661) and duplications (*λ* = 0.347). Genome-wide threshold was set to *p* < 1.65E-05 for deletions and *p* < 9.63E-06 for duplications (Bonferroni correction).

We identified 7177 of the 28,295 genes tested (25.4%) to be overlapped by CNV calls. We did not find genome-wide CNVs associated with dementia in this population, both in deletions and duplications using linear regression (Bonferroni-corrected *p* = 1.65 × 10^−05^ and 9.63 × 10^−06^, respectively, Fig. 1). However, we observed nominal deletions-associations with dementia at chr11, overlapping *PKP3* (median = 93.9 kb, *p* = 1.05 × 10^−03^) and *SIGIRR* genes (median = 98.7Kb, *p* = 2.23 × 10^−03^), as well as at chr12 overlapping *FBRSL1* gene (median = 97.2 kb, *p* = 2.18 × 10^−04^, Supplementary Fig. 1). Although these associations did not exceed the multiple testing threshold, the regions identified may represent promising loci for follow-up in larger cohorts or functional studies.

### Case-only CNV findings

We identified 2970 genes across the genome that were overlapped by CNV calls exclusively in dementia cases, with no overlaps observed in controls from the GR@ACE/DEGESCO study. Genes overlapped by CNV calls from at least four independent cases are shown in Supplementary Table 4, suggesting that they may represent structural abnormalities with potential functional relevance to dementia pathogenesis, though replication in independent cohorts will be necessary to confirm their role. Pathway enrichment analysis of these genes highlighted significant involvement in cardiac muscle adaptation, hypertrophic signaling, and vascular response pathways, indicating that these CNVs may influence disease risk through mechanisms involving cardiovascular remodeling and dysregulation (Supplementary Table 5).

A duplication encompassing the 14q11.2 region was identified exclusively in AD patients (*n* = 8) and was absent in controls (Supplementary Table 6). This duplication is located on chromosome 14 and fully spans the *CMTM5, EFS, IL25, MHRT, MIR208A, MIR208B, MYH6, MYH7* genes, extending both upstream and downstream in all cases, with an estimated size ranging from 78.7 to 146.1 Kb (Supplementary Fig. 2). Among the genes encompassed, *MYH6* and *MYH7* are the only ones with well-established pathogenic variants in ClinVar, primarily associated with hypertrophic and dilated cardiomyopathy^31,32^. Both encode cardiac myosin heavy chains and rank among the most frequently implicated genes in inherited cardiomyopathies. To assess whether duplication carriers also harbored co-occurring pathogenic point mutations at this locus, we examined ClinVar-annotated pathogenic, likely pathogenic, and conflicting pathogenicity variants in *MYH6* and *MYH7* (*n* = 98; *MYH6* *n* = 5, *MYH7* *n* = 93). Of these, only three variants were available in the TOPMed imputation panel with high quality (Rsq > 0.3), and none were present in any of the duplication carriers. This indicates that the potential cardiac co-pathology associated with this locus is driven by the copy number gain itself rather than by co-occurring pathogenic point mutations in *MYH6* or *MYH7*.

**Fig. 2:**
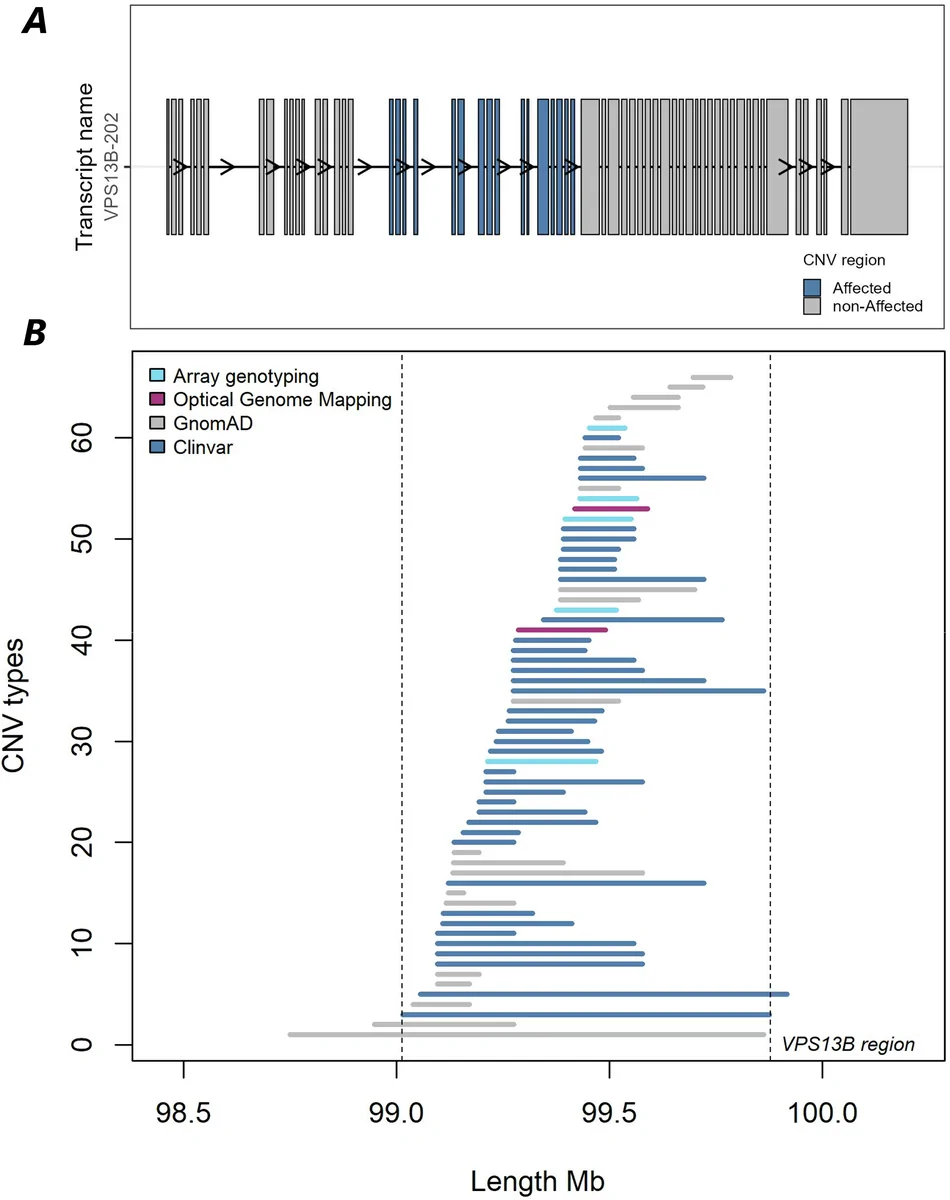
*VPS13B* gene region. **A** Mane transcript visualization (VPS13B-202 or ENST00000357162; 62 exons). Region affected by the CNVs found in GR@ACE/DEGESCO cohort is highlighted in red (exons 18–33). **B** Different deletions detected in the *VPS13B* gene region (genomic positions in GRCh38/hg38 assembly). Array genotyping and optical genome mapping correspond to the GR@ACE/DEGESCO individuals, whereas Clinvar and GnomAD correspond to open databases.

We also observed intragenic deletions affecting the Vacuolar Protein Sorting 13 Homolog B gene (*VPS13B*) in chromosome 8 in nine dementia patients. Deletions occur quite uniformly in all cases, affecting 82–254.8 Kb range and exons 18–33 out of 62 (Fig. 2, Table 2).

**Table 2:** Dementia patients with deletions in *VPS13B* gene found by genotyping array and/or optical genome mapping technology (OGM)

Indiv.	CNV start	CNV end	Nsnps	Length (kb)	Technique	Age	Sex	Phenotype
1	chr8:99466383	99586493	16	120.1	Array	68.6	Male	FTD
1	chr8:99418180	99598117	--	177.9	OGM	68.6	Male	FTD
2	chr8:99429591	99564413	20	134.8	Array	88	Female	AD
3	chr8:99452443	99586493	17	134.1	Array	84.7	Male	AD
3	chr8:99418180	99598117	--	177.9	OGM	84.7	Male	AD
4	chr8:99374974	99515703	22	140.7	Array	75	Male	AD
5	chr8:99429591	99564413	20	134.8	Array	89	Male	AD
6	chr8:99452443	99535165	10	82.7	Array	91	Female	AD
7	chr8:99212992	99467786	32	254.8	Array	74.7	Male	AD
8	chr8:99395405	99550235	22	154.8	Array	73	Female	AD
9	chr8:99338516	99467786	20	129.3	Array	84.4	Female	AD
9	chr8:99285332	99489787	--	196.0	OGM	84.4	Female	AD

Positions were in genome assembly GRCh38/hg38. *FTD* frontotemporal dementia, *AD* Alzheimer’s disease.

**Fig. 3:**
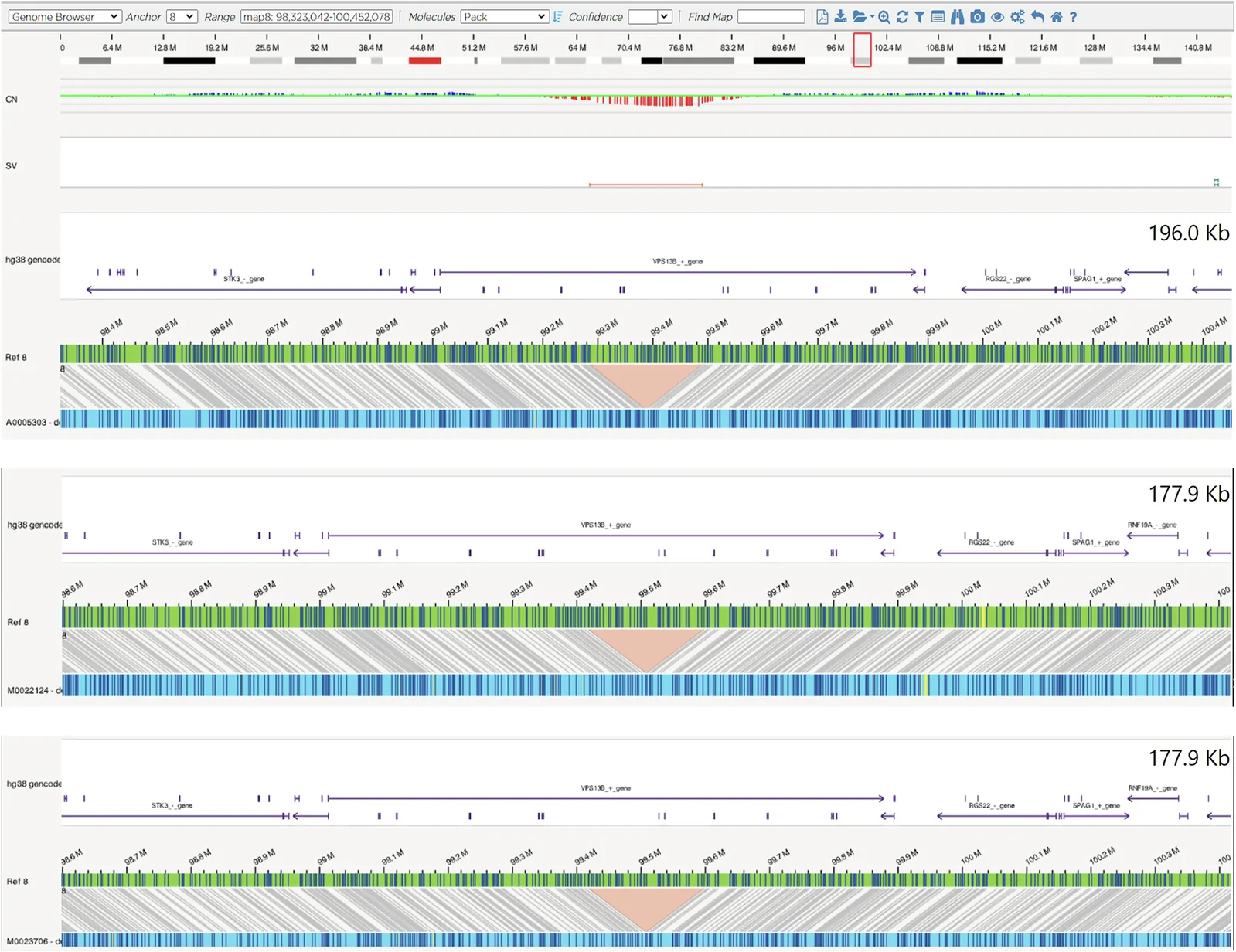
*VPS13B* CNV regions of three individuals from GR@ACE/DEGESCO by optical genome mapping. Direct visualization of structural variants was done on Bionano Access v1.7.1. using Genome Reference Consortium GRCh38/hg38 as the reference.

OGM enabled detailed molecular characterization of the CNVs in the *VPS13B* gene. Direct visualization of molecules revealed a rearrangement in this genomic region in all samples tested (*n* = 3, Fig. 3). In all three samples, the region identified by the array is fully contained within the larger region detected by OGM. The latter expands the initial region by ~44%, suggesting a broader or more sensitive capture of the associated signal. In the case of individuals 1 and 3, age at onset 68.6 and 84.7, respectively, we observed a 177.9 Kb deletion on chr8:99418180–99589117 encompassing exons 22–33 from *VPS13B* gene. On the other hand, for individual 9, age at onset 84.4, the deletion covered a region of 196 Kb (chr8:99285332–99489787) and affected exons 20–25 (Table 2). We therefore sought to further investigate the mutations of this gene.

In ClinVar, 3681 entries were identified for the *VPS13B* gene, with 512 being CNVs, constituting 13.9% of the total. Among these CNVs, 63% were classified as pathogenic or likely pathogenic. Specifically, 76% of the CNVs were linked to Cohen Syndrome–rare autosomal recessive disorder caused by biallelic loss-of-function variants in *VPS13B*–while the majority of the remaining 24% comprised unspecified cases and other pathologies. Approximately, 10% of the CNVs (*n* = 51) overlapped with the region affected in our individuals, with 43 (84.3%) deletions, of which 32 (74%) were referred to the condition of Cohen syndrome (Supplementary Table 7). Furthermore, it seems that all deletions we have detected bear striking similarity to those previously described as uncertain, pathogenic or likely pathogenic in ClinVar or GnomAD (Fig. 2). In addition, 668 SNPs were found in the TOPMed imputation reference panel, with a minor allele frequency (MAF) < 1%, classified as uncertain, pathogenic or likely pathogenic, from which only 169 of these variants met the high quality (Rsq > 0.3). We found two SNPs significantly associated with Cohen’s Syndrome (conflicting pathogenicity) for AD (rs141637316(G), OR = 3.85[1.01-14.65], *p* = 0.0487; and rs142248228(A), OR = 3.93 [1.01–15.29], *p* = 0.0499). Although Poisson regression analysis suggested a nominally significant increase in variant counts in AD cases (*p* = 1.52 × 10^−03^), this effect was not significant using models accounting for overdispersion (overdispersion ratio = 6.83; NBR OR = 1.02 [0.98–1.05], p = 0.334) or non-parametric testing (*p* = 0.282), suggesting limited burden difference between groups (Supplementary Fig. 3). When we examined those ClinVar-annotated SNPs across the nine deletion carriers, only one carrier harbored a single heterozygous variant of conflicting pathogenicity (chr8:99778930:G:A, GRCh38/hg38 assembly). This variant showed comparable frequency between cases and controls in the full cohort (1.33% vs 1.41%), suggesting that the identified deletions represent independent structural events rather than part of a broader mutational load at this locus.

### Candidate gene approach (AD genes)

We identified 45 of the 76 candidate genes tested (59%) that overlapped with CNV calls in the GR@ACE/DEGESCO study (Supplementary Table 8). Global AD burden analyses did not reveal significant differences between dementia cases and controls in CNV rate, distribution of deletions or duplications (deletions Fisher test *p* = 0.0875, duplications Fisher Test *p* = 0.861). However, we identified a deletion affecting the *MAPT* gene in 14 individuals, including 13 controls and one dementia case (Freq_ctrl-dem_ = 0.16–0.01%, Supplementary Fig. 4). Notably, all carriers shared the H2 haplotype (H1 = 49.4%, H1/H2 = 41.7%, H2 = 8.85% in GR@ACE/DEGESCO control population), which has been associated with reduced MAPT expression and decreased risk for tauopathies^33^. In contrast, risk-associated *ABCA7* deletions (four controls and 11 cases, Freq_ctrl-dem_ = 0.05–0.14%, *p* = 4.7 × 10^−03^) and *APP* CNVs were observed (three dementia cases, Freq_dem_ = 0.038%; deletions = 2, duplication = 1, Supplementary Table 9).

### ClassifyCNV

A total of 19,734 unique CNVs were identified in AD and control individuals (54.2% deletions). Among these, only 269 CNVs (1.36%) were classified as pathogenic or likely pathogenic, while the majority, 17,517 CNVs (88.8%), were of uncertain significance.

The untargeted analysis of pathogenic deletions (*n* = 243, 90.3%), based on the full CNV region, did not yield any statistically significant association. The strongest signal was observed in the *MAPT*-*STH*-*KANSL1* locus (chr17:44071294:44358286, GRCh37/hg19), with three occurrences in control subjects and none in AD cases (Fisher’s *p* = 0.115). Similarly, the sensitive gene-based approach identified four genes with nominal Fisher’s *p* values < 0.05 (*KANSL1*, *COL5A1*, *NOTCH1* and *PKD1*), although none remained significant after Bonferroni correction.

## Discussion

CNVs have emerged as relevant contributors to neurodegenerative disorders, yet their role in dementia remains uncertain. Some studies have investigated CNVs and other structural variants in different forms of dementias^6,8,20,29,30,34–39^, but their pathogenicity is often difficult to establish, particularly in the absence of segregation evidence, making it unclear whether they act as primary drivers or modifiers of disease risk. While CNVs involving well-known disease-associated genes can be linked more readily to disease mechanism, interpreting the significance of CNVs in genes without established disease-causing mutations remains particularly challenging.

In this study, by analyzing CNVs in a large Spanish cohort of array-genotyped individuals with detailed clinical and diagnostic information, enabled us to identify potentially novel CNV-affected genes associated with dementia and confirm CNVs as a source of genetic risk for dementia. Differences in CNV burden across studies—including ADNI^16^, NIA-LOAD/NCRAD^40^, TGen^8^ and AD Sequencing Project^39^—likely reflect variation in study design, genotyping platforms, QC procedures, or cohort composition, underscoring the need for harmonized analytical strategies^41^.

Our candidate AD-gene approach revealed CNVs in *APP* and *ABCA7* genes, supporting the potential role of these CNVs in the pathogenesis of AD. In contrast, partial and total deletions affecting the *MAPT* locus were observed almost exclusively in controls, and all carriers harbored the H2 haplotype. The H2 haplotype is known to contain a large inversion^42,43^ ( ~ 900 kb) and has been linked to reduced risk for several tau-related disorders^33,44–49^, including AD^50–52^. SV on the H2 background is less frequent than on H1^53^, yet the presence of deletions in this context may reflect a stable, inherited structural variant with a potential protective effect. Alternatively, it could point to a regional *MAPT* effect, given that the H2 haplotype is more common in the Spanish population^54^. Since *MAPT* dosage and expression levels are tightly linked to tau pathology, it is plausible that reduced copy number in the context of H2 might contribute to resilience against neurodegeneration. Additional CNVs in the *MAPT–STH–KANSL1* region were also highlighted by the classifyCNV approach, a tool for prioritizing potential pathogenicity. However, these variants appeared more frequently in controls and did not reach statistical significance, suggesting they may represent benign SV with context- specific effects unrelated to AD or limited functional relevance. Functional studies will be required to explore this hypothesis further, the consequences of these deletions and their potential role in modulating disease susceptibility, gene expression and disease progression.

Interestingly, two of our top candidate genes in the genome-wide approach, *PKP3*^55^ and *FBRSL1*^56^, have potential functional connections to neurodevelopmental delay and related conditions such as ADRD. *PKP3* (Plakophilin 3) is a gene encoding a protein involved in cell adhesion, particularly in maintaining the structural integrity of tissues^57,58^. It’s primarily associated with diseases such as arrhythmogenic right ventricular cardiomyopathy and certain skin disorders^59^. Its related pathways include the nervous system development. On the other hand, *FBRSL1* (Fibrinogen Receptor Like 1) encodes a integrin-related protein involved in cell adhesion and signaling. Pathogenic variants in this gene have been associated with an intellectual disability syndrome characterized by developmental delay, autistic traits, and multiple systemic features^60–64^. Furthermore, expression data from GTEx and cloning studies indicate moderate to high FBRSL1 expression across adult brain regions, with highest levels in cerebellum and hippocampus^56,65^.

Our third candidate, *SIGIRR* (also known as IL-1R8 or TIR8), is a negative regulator of innate immune signaling expressed in neurons, astrocytes and microglia^66–68^. It inhibits TLR and IL-1R pathways^69^, thereby attenuating IL-1β-mediated inflammation. In mice, SIGIRR deficiency leads to increased IL-1β signaling, impaired synaptic plasticity, and cognitive deficits, highlighting its potential role in modulating neuroinflammation and cognition^70^. Given the chronic upregulation of IL-1β and related cytokines in AD, the SIGIRR’s involvement in the anti-inflammatory IL-37 pathway, and the IL-1β-IL-1R axis has been implicated in causing tau pathology in AD. Loss-of-function or deletion of *SIGIRR* could exacerbate neuroinflammation and contribute to AD pathogenesis^71,72^.

Our study identifies genes enriched in cases relative to controls, suggesting the possible involvement of these genes in AD and/or dementia susceptibility. While the small number of carriers limits statistical power, the absence of these CNVs in controls raises the possibility of a potential disease-specific effect. For example, the duplication in the 14q11.2 region enriched in cases, encompassing multiple genes including *IL25*, a regulator of neuroinflammatory responses in neurodegenerative diseases^73^. Elevated IL25 levels have been associated with cognitive decline, indicating its potential as a biomarker for disease progression^74^. Given that the CNV also spans other genes ( ~79–146 Kb), the biological and clinical relevance of this duplication likely extends beyond *IL25* alone and needs replication. Notably, among the duplicated genes, *MYH6* and *MYH7* encode cardiac myosin heavy chains and are the primary genetic causes of inherited hypertrophic and dilated cardiomyopathies^31,32^. Increased dosage of these genes may alter myosin stoichiometry and contribute to cardiac co-pathology, a finding that is strikingly consistent with the enrichment of vascular and cardiac remodeling pathways observed in our case-only CNV pathway analysis. This raises the possibility of a contiguous gene effect, where the combined dosage changes of multiple neighboring genes—spanning neuroinflammatory (*IL25*) and cardiac (*MYH6, MYH7*) functions—may jointly contribute to the dementia phenotype through convergent vascular and inflammatory mechanisms. Functional characterization of all affected genes will be necessary to clarify the biological relevance and pathogenic potential of this duplication.

Notably, this is the first report describing intragenic deletions in *VPS13B* among ADRD cases (*n* = 9, Freq_cases_ = 0.115%), validated by an independent technique (OGM). These deletions span exons 18–33 out of 62, removing a substantial portion of the coding region and likely disrupting protein structure and function—either by causing a frameshift and premature termination (leading to nonsense-mediated decay) or by deleting critical domains involved in vesicular trafficking and Golgi organization.

Loss-of-function mutations in *VPS13B* are known to cause Cohen syndrome^75,76^, characterized by phenotypic consequences including developmental delay, intellectual disability, microcephaly, hypotonia and systemic features, which supports a potential deleterious effect of these variants on neuronal homeostasis, although their relevance to dementia requires further investigation. While the GnomAD CNV database lists 20 types of deletions affecting *VPS13B* gene region (*n* = 108 in 464,297 individuals, Freq = 0.023%, Fig. 2), no previous experimental or genetic studies have linked this locus to ADRD. However, additional evidence emerges from two rare pathogenic SNPs in *VPS13B* (rs141637316 and rs142248228) previously linked to Cohen syndrome, which also showed significant associations with AD. The convergence of structural and rare variant signals, together with reported associations with AD-related endophenotypes such as hippocampal volume^77^, further support its candidacy as a dementia susceptibility gene.

Strikingly, mutations in other VPS13 family members are also implicated in neurological disorders, including chorea-acanthocytosis (VPS13A)^78^, syndromic autism^79–81^ (VPS13B), early-onset Parkinson’s disease (VPS13C)^82,83^ and ataxia (VPS13D)^84^. This diversity highlights the broad role of VPS13 proteins in brain health. *VPS13B* encodes a large transmembrane protein likely involved in vesicle-mediated transport and protein sorting^85^. Disruptions in lipid transport at membrane contact sites—where VPS13 proteins operate—are linked to several neurological disorders, emphasizing the importance of organelle lipid homeostasis for neuronal function^86,87^. Additionally, *VPS13B* plays a role in nervous system development and may be essential for neuron projection growth^88,89^.

Furthermore, pathway analysis in the duplications exclusively in dementia cases revealed the presence of well-established roles in cardiac and vascular biology, suggesting a potential link to vascular co-pathology^90,91^ in AD. These findings may reflect a shared genetic vulnerability affecting both cardiac and cerebral vascular systems, potentially contributing to blood-brain barrier breakdown, neuroinflammation, and impaired perfusion, all of which are common in patients with mixed or vascular-influenced dementia^92,93^.

Despite the progress in understanding CNVs in dementia, significant challenges remain for clinical translation. The heterogeneity of CNVs and their interaction with genetic, epigenetic, and environmental factors complicate the definition of clear mechanistic hypotheses, underscoring the need for integrative and multidisciplinary approaches. We also acknowledge the limitations of array-based CNV detection. While arrays provide a cost-effective genotyping of large cohorts, their resolution depends on marker density^94^, leading to conservative estimates and reduced sensitivity in poorly covered regions, such as near centromeres. Moreover, arrays cannot resolve breakpoints at base-pair resolution or differentiate structural events like inversions or translocations, and the detection of small CNVs ( < 1 kb) remains particularly challenging due to signal variability. Additionally, as is typical of population-based dementia cohorts, cases and controls usually differ in age and sex distribution; sex-stratified and age-matched analyses in larger cohorts would be valuable to assess whether these factors differentially influence CNV distribution and association results. Furthermore, replication of rare CNV associations presents a fundamental challenge in the field, given the low frequency of individual structural events. This underscores the broader need for large-scale collaborative initiatives and harmonized analytical frameworks that enable the aggregation of CNV data across multiple cohorts. Encouragingly, the independent experimental validation of the most prominent findings—specifically *VPS13B* intragenic deletions confirmed by optical genome mapping—provides orthogonal structural evidence that strengthens confidence in the biological relevance of these loci beyond what array-based detection alone could offer.

The present work represents the first CNV analysis within the GR@ACE/DEGESCO cohort, the largest clinically characterized dementia cohort in Spain. We identified deletions in *VPS13B, PKP3*, *FBRSL1* and *SIGIRR* genes and duplications in 14q11.2 region, suggesting possible contributions to ADRD, emphasizing the importance of further research to elucidate their underlying mechanisms. As evidence linking CNVs and ADRD grows, genotyping array data remains valuable for identifying structural variants with potential pathogenic roles. Nonetheless, complementary strategies such as long-read sequencing, functional characterization, and standardized meta-analyses will be essential to replicate findings and fully leverage the potential of structural genomics to improve diagnosis, prognosis, and treatment of dementia, thus offering hope for better outcomes for people affected by these complex neurodegenerative conditions.

## Methods

### GR@ACE/DEGESCO samples

The individuals who participated in the study were obtained from multiple Spanish centers collected by GR@ACE/DEGESCO consortium. The GR@ACE cohort recruited individuals across the spectrum of cognitive decline associated with dementia, including those with mild cognitive impairment (MCI) and established dementia diagnoses, as originally defined in the GR@ACE study^90,95^ design from Ace Alzheimer Center Barcelona (Barcelona, Spain), and control individuals from three centers: Ace Alzheimer Center Barcelona, Valme University Hospital (Seville, Spain), and the Spanish National DNA Bank Carlos III (University of Salamanca, Spain) (http://www.bancoadn.org). Additional dementia, MCI cases and population healthy controls were obtained from 13 research centers included in Dementia Genetic Spanish Consortium (DEGESCO)^96^.

At all sites, AD diagnosis was established by a multidisciplinary working group—including neurologists, neuropsychologists, and social workers—according to the DSM-IV criteria for dementia and the National Institute on Aging and Alzheimer’s Association’s (NIA–AA) 2011 guidelines for diagnosing AD. For MCI diagnosis, the classification of López et al.^97^ and Petersen’s criteria were also used^98–100^. For further details^5,90,95^ on the contribution of the sites, see Supplementary Table 1. Written informed consent was obtained from all the participants. The ethics and scientific committees have approved this research protocol (Acta 25/2016, Ethics Committee H., Clinic I Provincial, Barcelona, Spain). All procedures were conducted in accordance with the principles of the Declaration of Helsinki.

Methods for genetic data acquisition and detailed quality control (QC) procedures have been previously described^90,95^. Genotyping was conducted using the Axiom 815 K Spanish biobank array (Thermo Fisher) at the Spanish National Center for Genotyping (CeGEN, Santiago de Compostela, Spain). SNP-arrays are used to evaluate the intensity of the hybridization signal of genomic DNA with the average value of control DNA using single-source hybridization instead of competitive hybridization, being able to detect submicroscopic CNVs^101^.

### CNV detection and quality control

CNV calls were generated using PennCNV^102^ v.1.0.5 on raw signal intensity data from each genotyping array. The PennCNV-Affy protocol (http://www.openbioinformatics.org/penncnv/penncnv_tutorial_affy_gw6.html) was first performed to transform raw CEL files into a signal intensity file containing the Log R Ratio (LRR) as a normalized measure of the total signal intensity for two alleles of the SNP and the B allele frequency, which is a normalized measure of the allelic intensity ratio of two alleles to generate CNV calls. We followed the user guidelines of PennCNV^102^ and used the Hidden Markov Model^102,103^ “affygw6.hmm” and a population frequency of B allele (PFB file) calculated with the coordinates from the Axiom SpainBA array. The combination of LRR and BAF was used to determine the presence of CNVs in the genome^104,105^ and we extracted all duplications and deletions covering the genome (HG build 37).

Extensive QC of CNV calls was performed on all samples, restricting the analyses to autosomes (Supplementary Table 2). Adjacent CNVs were merged where the gap was <30% of the total length (--fraction 0.3). Quality metrics included CNV call counts, LRR standard deviation (SD), BAF Drift and Waviness Factor (WF). Samples were excluded if they had an excessive number of CNVs ( > 50 CNV) and exceeded any of the following thresholds: LRR SD > 0.38, BAF Drift>0.015 or WF > 0.1. To ensure we were including only high-confidence CNVs in the analysis, CNVs called based on data <10 SNPs, length <50 kb and CNVs that had >50% overlap with chromosomal centromeric and telomeric, or immunoglobulin regions (as defined in Need et al.^106^) extended by 250 kb on each boundary were excluded as they are known to be prone to artificial CNV calls and therefore false discoveries.

Individuals with low-quality SNP raw data, excess of heterozygosity, sex discrepancies and familial relations between samples (PI-HAT > 0.1875) were excluded from the analysis. A principal component analysis (PCA) was performed, and population outliers were removed (European cluster of 1000 Genomes). Additionally, individuals with Down Syndrome were excluded from the analyses due to trisomy chr21 and the high probability of structural DNA alterations.

CNVs may involve one or more genes and are distributed in a non-uniform manner. In this sense, we annotated the overlapping genes (partially or totally) by each CNV using the reference gene annotation (refGene.txt.gz and refLink.txt.gz) from UCSC Genome Browser build 37. To minimize potential batch effects related to CNV calling, we performed an additional PCA based on the presence/absence of annotated gene deletions and duplications. Individuals falling outside ±3 SD from the mean of PC1 were excluded as potential outliers. Subsequently, individuals carrying any of the 15 genes indicated by Aguirre et al.^29^ based on data from UK Biobank, classified as genes with greater intolerance to SV (*BRCA2, BRCA1, APC, ATM, MSH2, MLH1, MSH6, RB1, SBDS, SPATA31D1, CYP3A4, PABPC3, OTOP1, KRT16* and *ZNF302*), were excluded. These genes were enriched in early-onset diseases, particularly cancer^29,107–109^. Afterwards, to avoid batch effects, analysis of variance (ANOVA) comparisons between ctrl-ctrl individuals based on different sources of DNA origin (i.e. coming from extracted DNA, blood, saliva or DNA banks) were performed in duplicates and deletions separately by removing genes with *p* value < 0.001. Finally, 16,093 samples (7818 dementia cases and 8275 controls) with 43,833 CNV calls remained after all QC measures and were entered into case/control association analyses (Supplementary Table 2).

Comparisons between dementia cases and controls were performed using the Wilcoxon rank-sum test for continuous variables (e.g., CNV count, length, genes), and chi-square tests for categorical comparisons (CNV presence and sex distribution). All analyses were conducted in R (version 4.4.1), p values < 0.05 were considered statistically significant.

### CNV association analyses

Gene-level dementia case/control association tests were conducted across all genes in 16,093 unrelated individuals of Spanish ancestry using linear regression implemented in Plink2^110^ (www.cog-genomics.org/plink/2.0/), including the 4PCs based on the annotated gene CNVs as covariates. Gene burden was encoded as a binary variable, which indicates whether an individual has a CNV which contains any overlap of the HUGO Gene Nomenclature Committee gene region. For CNVs spanning multiple genes, each affected gene was individually considered in the analysis. We treat deletions and duplications identically, with the assumption that any CNV which overlaps a gene in this fashion will disrupt its normal function. Genes were considered separately for deletions (*n* = 3035) and duplications (*n* = 5192) in the genome-wide approach, and Bonferroni correction for multiple testing was applied accordingly for each CNV type (0.05/3035 = 1.65 × 10^−05^ for deletions and 0.05/5192 = 9.6 × 10^−06^ for duplications). Another case-only analysis was performed to identify genes that may play a role in dementia susceptibility using the Fisher Test.

The quantity and quality of CNVs reported by different algorithms and different platforms exhibit substantial variance; therefore, it is often recommended to visually examine CNV calls to judge whether they are reliable or not. Candidate CNVs were visually inspected with R software (4.4.2) using the BAF and LRR values.

### CNV burden analyses

To determine whether dementia cases show a greater number of CNVs (deletions and duplications), a *t* test was performed. Furthermore, burden analysis (Fisher Test) of AD genes affected by CNVs was conducted between dementia cases and controls, including 76 AD genes identified from the latest meta-GWAS by the European Alzheimer’s and Dementia consortium^5^.

### Optical genome mapping

OGM^111,112^ is a high-resolution genomic analysis technique that offers a comprehensive view of an individual’s DNA structure. This cutting-edge technology uses advanced imaging of very long linear single DNA molecules (median size > 250 kb) and computational methods to map their arrangement within the genome. Thus, OGM was used for an independent experimental validation of CNVs in the *VPS13B* gene regions of the individuals for whom we had blood availability (*n* = 3 out of nine samples).

#### DNA extraction, labeling, and chip loading

For each sample, ultra-high molecular weight genomic DNA (gDNA) was extracted from 650 μL of frozen blood following manufacturer’s protocols (Bionano Prep SP Blood and Cell DNA Isolation Kit; Bionano Genomics, San Diego, CA). Briefly, after thawing in 37 °C water bath, white blood cells were counted, pelleted (2200 × *g* for 2 min), and treated with proteinase K (Qiagen) and LBB lysis buffer to release gDNA. After phenylmethylsulfonyl fluoride treatment (Sigma-Aldrich, St. Louis, MO) to inactivate proteinase *K*, a Nanobind paramagnetic disk was added to the solution, mixed with isopropanol to precipitate the gDNA and washed with WB1 and WB2. After transferring to a new tube, buffer EB (Bionano Genomics, San Diego, CA) was added to elute the gDNA from the disk. The gDNA was equilibrated overnight at room temperature for homogenization and subsequently quantified with Qubit Fluorometer (Invitrogen™ Qubit™ 4 Fluorometer).

Sequence-specific direct label and stain (DLS) technique was used for labeling following manufacturer’s protocols (Bionano Prep DLS Labeling Kit; Bionano Genomics, San Diego, CA). Direct Labeling Enzyme 1 (DLE-1) reaction was carried out using 750 ng of gDNA to tag a specific 6 bp sequence (CTTAAG) with a DL-green fluorophore ( ~15 times per 100 kb). Following Puregene proteinase K (Qiagen, Hilden, Germany) digestion and DL-Green clean-up using DLS membrane in DLS 24-well plate, the labeled gDNA was mixed with DNA Stain, stained overnight at room temperature for backbone visualization and quantified using Invitrogen™ Qubit™ 4 Fluorometer. The fluorescent-labeled gDNA molecules (4–12 ng/µl) were loaded on a Saphyr chip G2.3, and linear double-stranded molecules passing across nanochannels were sequentially imaged on a Saphyr instrument (Bionano Genomics, San Diego, CA).

#### Data analysis and variant filtering

Effective genome coverage of at least 160× effective coverage was achieved for de novo assemblies. Standard run quality control parameters [total DNA (≥150 kbp), N50 (≥150 kbp), map rate (≥150 kbp), effective coverage (>300×), average label density (per 100 kbp)] were evaluated per manufacturer’s guidelines. Details regarding limit of detection, reproducibility and precision are described elsewhere^113,114^.

The de novo assembly (DN; SVs > 500 bp) pipeline was executed on Bionano Solve software v3.7. (Bionano Genomics, San Diego, CA). Reporting and direct visualization of structural variants was done on Bionano Access v1.7.1. using Genome Reference Consortium GRCh38/hg38 as the reference.

SVs were identified based on discrepancies in the labeling pattern between the sample and the reference. The complementary coverage-based CNV algorithm identified large chromosomal gains and losses (˃500 kbp). Confidence scores were calculated and provided by Bionano Genomics^113^. For data filtering, the following recommended confidence scores were used: Insertion/Deletion = 0; Inversion = 0.7; Duplication = −1; Intra-fusion/Inter-translocation = 0.05; Copy Number = 0.99; Aneuploidy = 0.95.

Genome imaging generated at least 550 Gb of data per sample later analyzed with the DN pipeline. Label density was 14.24 labels/100 kb on average. The average N50 molecule length (molecules ≥150 kb and at least nine labels) was 272.63 kb. Only molecules over 150 kb were included. Tall of this resulted in an average map rate of 79% and an effective coverage of 196.8× for DN pipeline (Supplementary Table 3).

### Post-hoc analyses

In the human genome, CNVs are also classified into several categories: benign CNV (normal genomic variant), likely benign CNV, variant of uncertain significance, CNV of possible clinical relevance (high-susceptibility locus/risk factor/likely pathogenic variant), and clinically relevant CNV (pathogenic variant). Information on the *VPS13B* gene region was extracted from ClinVar^115,116^, and comparisons were conducted to determine whether the identified CNVs had been previously described or appeared to be novel.

Regarding SNPs, all those reported in ClinVar with high imputation quality (Rsq>0.3) were extracted from the array, and an AD/Ctrl association was performed to assess whether other types of mutations affected the *VPS13B* region. Additionally, a burden analysis was conducted to evaluate the cumulative effect. Individual-level variant counts were calculated by summing heterozygous and homozygous minor alleles across all markers. Generalized linear models (GLMs), including Poisson and negative binomial regressions (NBR), and non-parametric testing (Wilcoxon) were applied to test for differences in variant burden between AD cases and controls. Distribution of variant burden per individual was visualized using violin and density plots.

The Genome Aggregation Database SV resource^117^ (GnomAD CNVs v4.1.0) was used as a reference for the types and frequencies of CNVs in the genes of interest within a larger cohort (includes 464,297 unrelated exomes). In addition, gene transcript information was extracted from this database. To enable cross-platform comparison between array-based, OGM results and current genomic databases (ClinVar, GnomAD), genomic coordinates for the *VPS13B* region were lifted over from GRCh37 to GRCh38 using the UCSC LiftOver tool, while all remaining array-based CNV coordinates are reported in GRCh37.

Furthermore, the ClassifyCNV^118^ software was employed for the accurate identification and clinical annotation of CNVs. This tool implements the 2019 ACMG classification guidelines to assess CNV pathogenicity using genomic coordinates and CNV type as input. It provides a clinical classification for each variant, along with a breakdown of the classification score and a list of genes potentially relevant for variant interpretation.

### Pathway analysis

Pathway enrichment analysis was conducted in R (4.4.2) using the genes affected by CNVs observed exclusively in at least four dementia cases. Gene annotations were converted to Entrez IDs, and both KEGG and Gene Ontology (GO, Biological Process) enrichment analyses were performed using the clusterProfiler() package. Significance thresholds were set at *p* value < 0.05, and *q* value < 0.2, with multiple testing correction applied using the Benjamini-Hochberg method.

## Supplementary information

Supplementary Information

202603_SupplementaryData_CNVs

## Data Availability

The data that support the findings of this study are available on request from the GR@ACE/DEGESCO consortium. Summary statistics of the CNVs by gene association analysis are publicly available at Zenodo (10.5281/zenodo.20026127).
