# Supplementary material for: Landscape of copy number variants in Spanish people with dementia
Source: NPJ Genom Med. 2026 Jun 30;11:58. doi: 10.1038/s41525-026-00589-6 (PMC13683362; doi:10.1038/s41525-026-00589-6)
Supplement: Supplementary file 1 [file 41525_2026_589_MOESM1_ESM.pdf]

Landscape of copy number variants in Spanish People with Dementia

Supplementary Figures

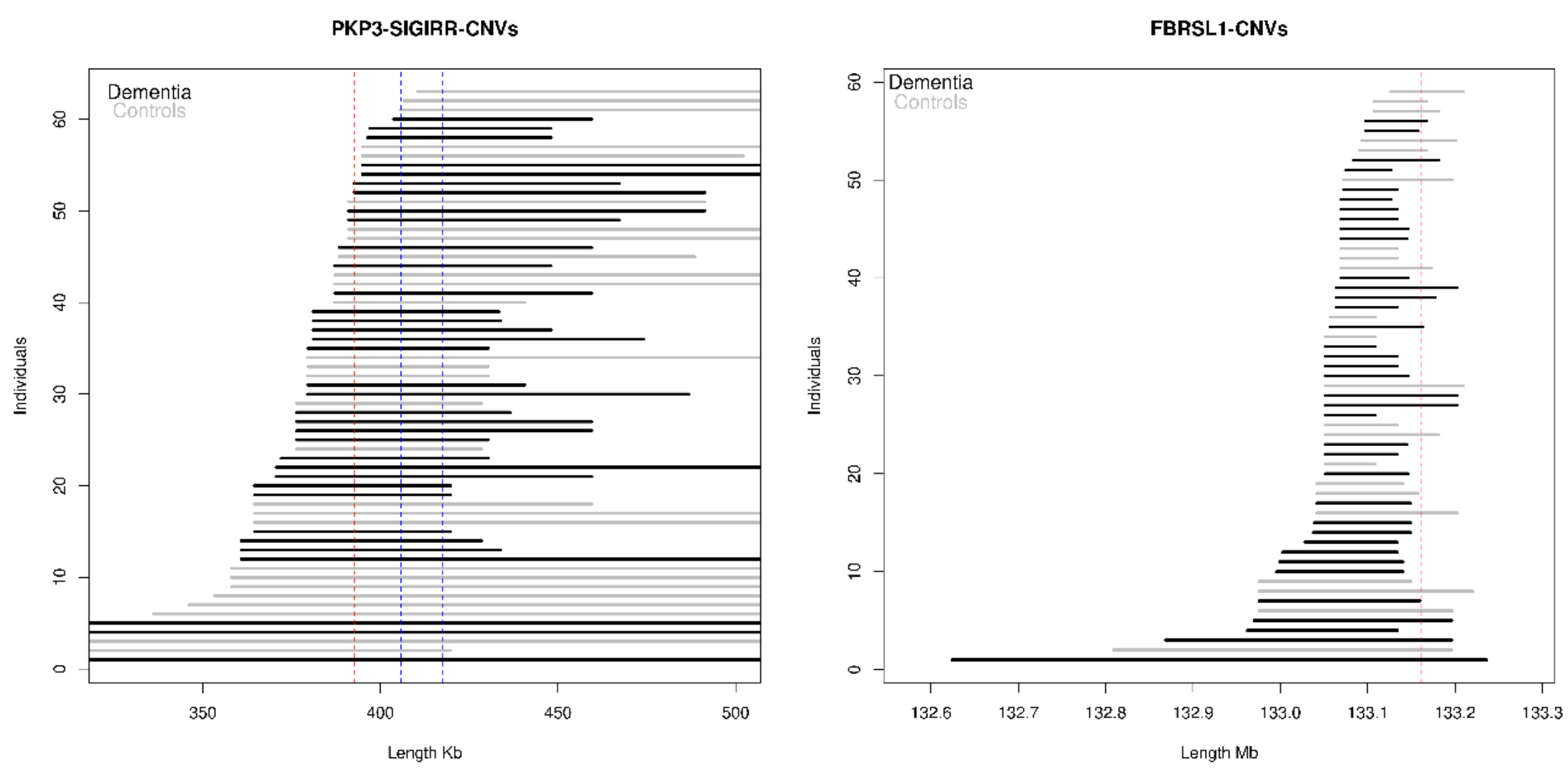

Supplementary Figure 1: Copy number deletions affecting the top genes suggestively associated with dementia. *PKP3* and *FBRSL1* gene regions were highlighted in red, *SIGIRR* gene region in blue. Positions were in genome assembly GRCh37/hg19.

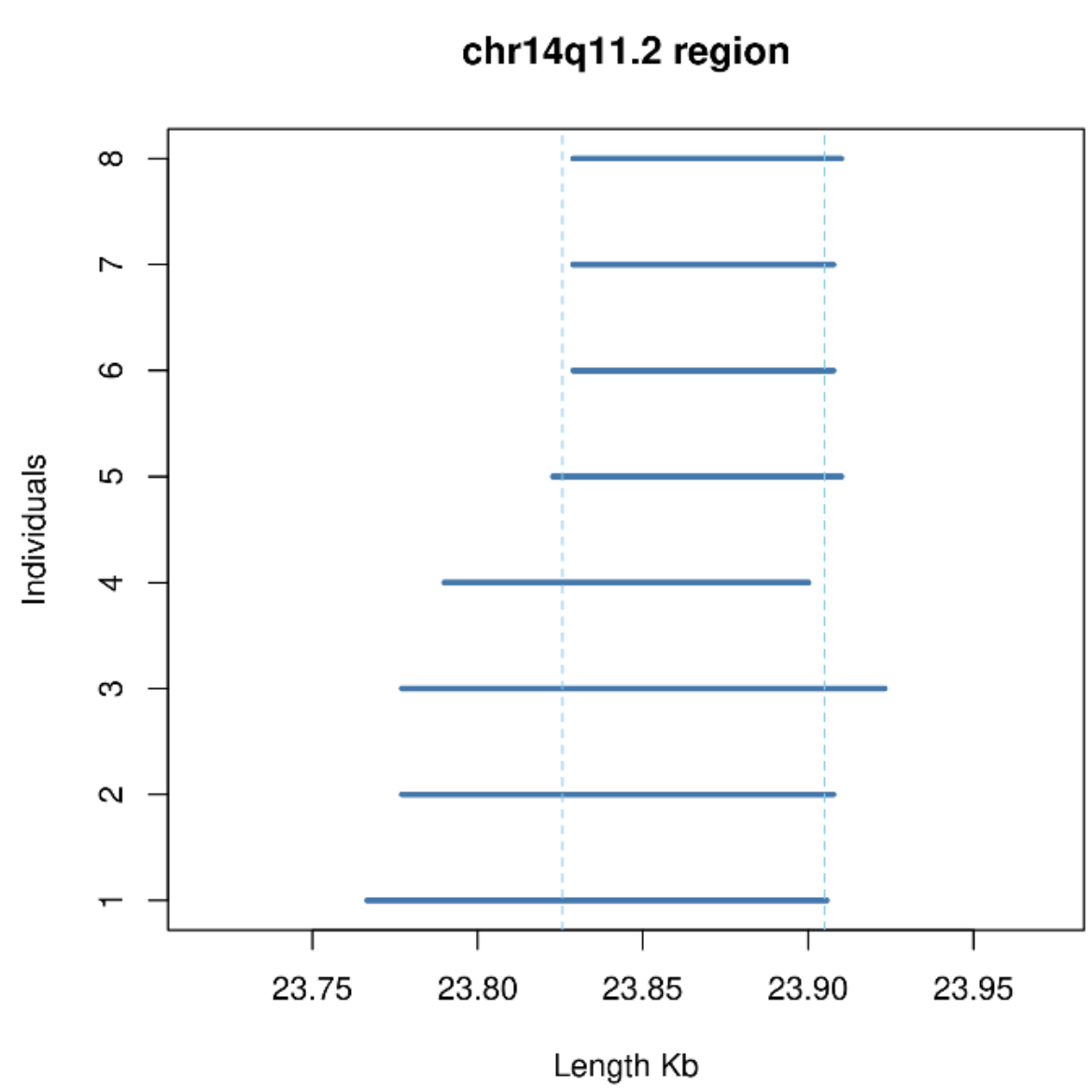

Supplementary Figure 2: Copy number duplications affecting the 14q11.2 region identified exclusively in dementia cases (see Supplementary Table 6). The shared genic region spanning *CMTM5*, *EFS*, *IL25*, *MHRT*,

MIR208A, *MIR208B*, *MYH6*, and *MYH7* is highlighted in skyblue. Positions are in genome assembly GRCh37/hg19.

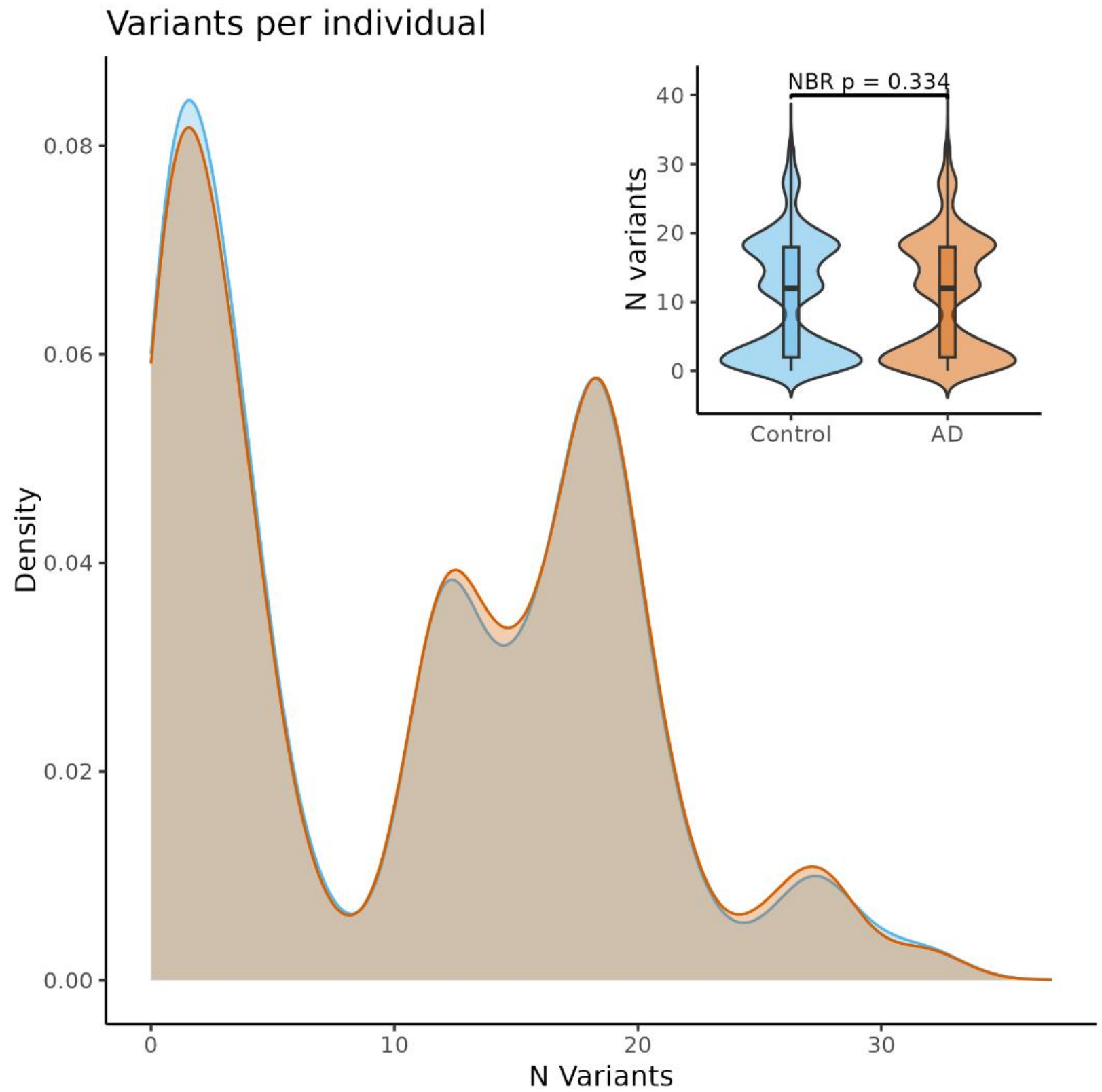

Supplementary Figure 3: Distribution of variant burden per individual in the *VPS13B* gene region for the 169 SNPs significantly associated with Cohen's Syndrome (ClinVar) available in the TOPMed imputation reference panel (Rsq>0.3, MAF<1%).

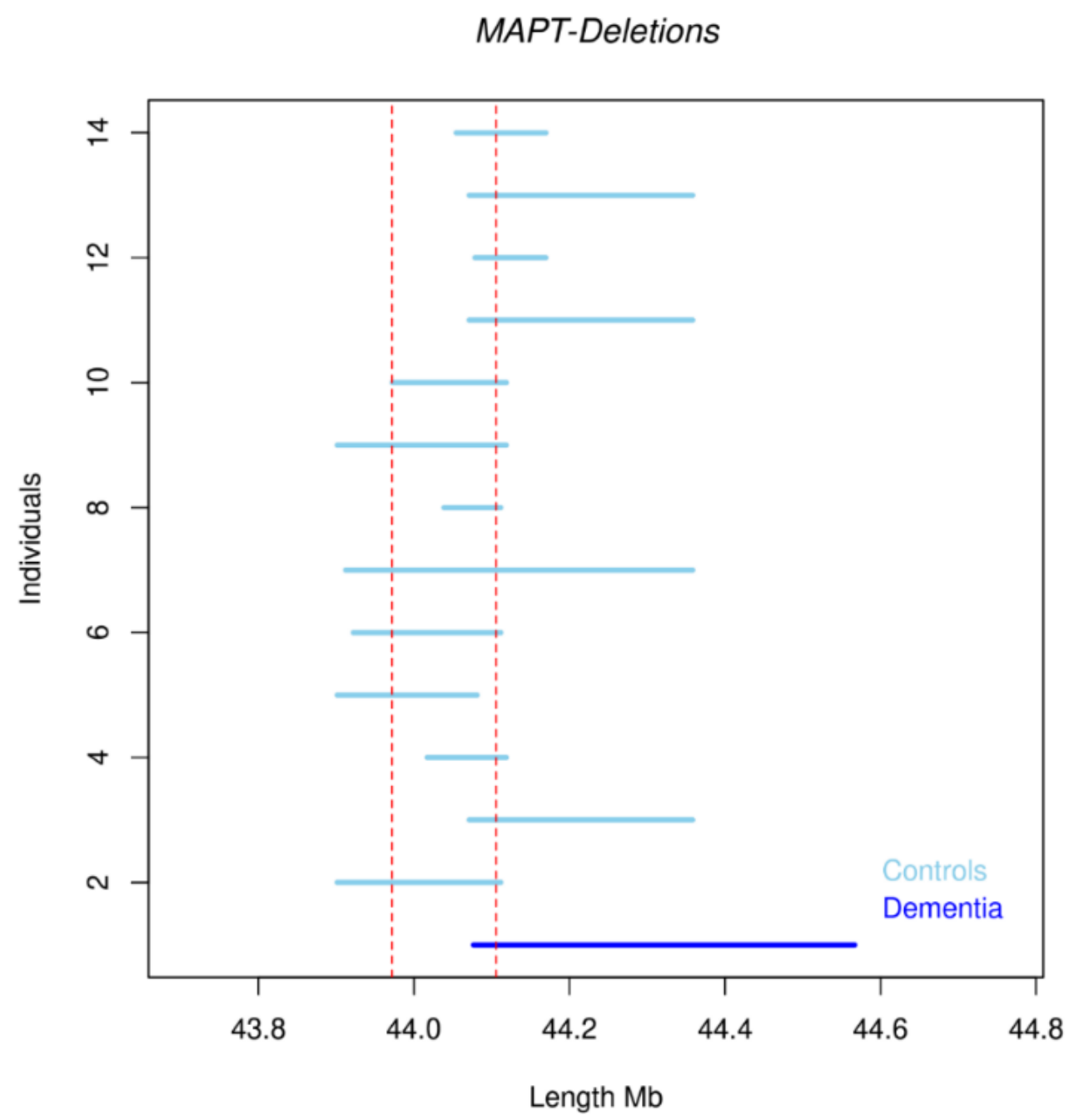

Supplementary Figure 4: Copy number deletions affecting the *MAPT* genomic region. Gene region were highlighted in orange. Positions were in genome assembly GRCh37/hg19.

Table of contents	
**Supplementary Table 1**	Description of datasets within GR@ACE/DEGESCO consortium.
**Supplementary Table 2**	Quality Control Steps for CNV Analysis.
**Supplementary Table 3**	Data analysis and variant filtering characteristics for the samples analyzed by optical genome mapping.
**Supplementary Table 4**	Case-Specific CNVs.
**Supplementary Table 5**	Pathway enrichment analysis of genes affected by CNVs exclusive to dementia cases.
**Supplementary Table 6**	Duplications identified in the 14q11.2 region.
**Supplementary Table 7**	ClinVar CNVs overlapping the VPS13B region identified in our cohort.
**Supplementary Table 8**	Counts of CNVs affecting Alzheimer's Disease-related genes.
**Supplementary Table 9**	CNV information for *MAPT* , *ABCA7* and *APP* Genes.

Supplementary Table 1. Description of datasets within GR@ACE/DEGESCO consortium.

Initial Sample

Number	*Center Name*	*Controls*	*AD*	*MCI*	*Other Dementia*	*Down Syndrome*	*Total*
1	Ace Alzheimer Center Barcelona (Barcelona, Spain)	2077	6533	821	467	11	9909
2	Spanish National DNA Bank Carlos III (Salamanca, Spain)	3680	297	0	0	0	3977
3	Alzheimer Center Fundación Reina Sofía (FCIEN) (Madrid, Spain)	1147	287	0	0	0	1434
4	Valme University Hospital (Seville, Spain)	1176	0	0	0	0	1176
5	Institute of Biomedicine of Seville (IBIS) (Seville, Spain)	877	183	0	0	0	1060
6	Biochemistry & Molecular Biology, School of Medicine, University of Malaga (Malaga, Spain)	292	141	0	0	0	433
7	Neurology Service, Marqués de Valdecilla University Hospital (Santander, Spain)	41	69	24	174	0	308
8	Mútua de Terrassa Research and Teaching Foundation (Barcelona, Spain)	103	161	0	0	0	264
9	Biobank, Institute for Biomedical Research (Lleida, Spain)	4	207	31	0	0	242
10	Central University Hospital of Asturias (Oviedo, Spain)	135	69	0	0	0	204
11	Ramón y Cajal University Hospital (Madrid, Spain)	0	39	0	0	0	39
12	Severo Ochoa Center for Molecular Biology, CBM-CSIC (Madrid, Spain)	8	16	0	0	0	24
13	Hospital de la Princesa (Madrid, Spain)	0	0	0	0	574	574
14	Biomedica Research Group, ALEXANDROS and GERO cohorts (Santiago, Chile)	289	131	1	2	0	423
	Total	9829	8133	877	643	585	20067

PostQC

Mild cognitive impairment, Down Syndrome and other populations were excluded in the quality control

Number	*Center Name*	*Controls*	*AD*	*MCI*	*Other Dementia*	*Down Syndrome*	*Total*
1	Ace Alzheimer Center Barcelona (Barcelona, Spain)	1587	5939	0	407	0	7933
2	Spanish National DNA Bank Carlos III (Salamanca, Spain)	3298	223	0	0	0	3521
3	Alzheimer Center Fundación Reina Sofía (FCIEN) (Madrid, Spain)	1034	253	0	0	0	1287
4	Valme University Hospital (Seville, Spain)	1012	0	0	0	0	1012
5	Institute of Biomedicine of Seville (IBIS) (Seville, Spain)	815	171	0	0	0	986
6	Biochemistry & Molecular Biology, School of Medicine, University of Malaga (Malaga, Spain)	257	139	0	0	0	396
7	Neurology Service, Marqués de Valdecilla University Hospital (Santander, Spain)	40	69	0	163	0	272
8	Mútua de Terrassa Research and Teaching Foundation (Barcelona, Spain)	97	148	0	0	0	245
9	Biobank, Institute for Biomedical Research (Lleida, Spain)	4	186	0	0	0	190
10	Central University Hospital of Asturias (Oviedo, Spain)	123	67	0	0	0	190
11	Ramón y Cajal University Hospital (Madrid, Spain)	0	38	0	0	0	38
12	Severo Ochoa Center for Molecular Biology, CBM-CSIC (Madrid, Spain)	8	15	0	0	0	23
	Total	8275	7248	0	570	0	16093

Supplementary Table 2. Quality Control Steps for CNV Analysis. This table summarizes the quality control steps applied during CNV (Copy Number Variation) analysis. It includes the criteria used at each step and the corresponding sample size retained after each filtering phase.*Mild cognitive impairment, Down Syndrome and other non-European populations were excluded (see methods)**.**

Quality control step	Criteria	Details	CNVs	Individuals with CNVs	Individuals
**Initial sample (n)**			198884	19877	20067
**Merge adjacent CNVs**	Merge	Gap <30% of total length	180882	19877	20067
**Filtering QC - CNVs**	Exclusion	LRR SD>0.38; BAF Drift>0.015;<br>WF>0.1;<br><10 SNPs; length <50kb	88169	19230	20067
**Telomers/centromers**	Exclusion	>50% overlap + 250Kb	83235	18151	20067
**Genotyping QC**	Exclusion	Het, sex, IBD, PCA, Mind,<br>Other phenotypes/populations*	71322	15710	17236
**CNVs QC**	Exclusion	PCA (+/-3SD)	65382	15475	17001
		nCNVs >50	53991	15372	16898
		Intolerant genes to CNVs	53885	15355	16881
		ANOVA DNA type (Ctrl/Ctrl)	45710	14904	16881
**Phenotype**	Inclusion	Dementia and controls	43833	14222	16093

Supplementary Table 3. Data analysis and variant filtering characteristics for the samples analyzed by optical genome mapping.
Mol=Molecules; Kb= Kilobase pair; Gb=Giga base pair; DN=De novo assembly.

Individual	Total DNA (Mol ≥ 150kb)	N50 (Mol ≥150 kb and min 9 labels)	Map Rate (%)	Estimated Effective Coverage	Average Label Density (per 100 kb)	Pipeline used
1	739.77 Gb	252.38 kb	71.5 %	171.27X	14.56	*DN*
3	1133.03 Gb	259.50 kb	72.6 %	258.64X	13.44	*DN*
9	553.1 Gb	306.00 kb	92.9 %	160.43X	14.71	*DN*

Supplementary Table 4. Case-Specific CNVs.

This table reports the number of duplications and deletions observed exclusively in case samples. Only genes with four or more CNV events in cases are included, providing insight into potentially relevant loci with higher CNV burden in the affected population.

	Duplications		Deletions						
**Individuals**	**CASE**	**Ctrl**	**CASE**	**Ctrl**	**FreqCNVs**	**FreqCNV_CASE**	**Gene**	**Phenotype**	**Type**
16093	3	0	2	0	0.0311	0.064	*APP*	DemvsCtrl	All CNVs
16093	2	0	3	0	0.0311	0.064	*BRWD1*	DemvsCtrl	All CNVs
16093	3	0	1	0	0.0249	0.0512	*BTG3*	DemvsCtrl	All CNVs
16093	3	0	1	0	0.0249	0.0512	*BTG3-AS1*	DemvsCtrl	All CNVs
16093	3	0	2	0	0.0311	0.064	*C21orf91*	DemvsCtrl	All CNVs
16093	3	0	2	0	0.0311	0.064	*C21orf91-OT1*	DemvsCtrl	All CNVs
16093	3	0	2	0	0.0311	0.064	*CHODL*	DemvsCtrl	All CNVs
16093	3	0	2	0	0.0311	0.064	*CHODL-AS1*	DemvsCtrl	All CNVs
16093	3	0	1	0	0.0249	0.0512	*HLCS*	DemvsCtrl	All CNVs
16093	3	0	1	0	0.0249	0.0512	*LINC01683*	DemvsCtrl	All CNVs
16093	3	0	2	0	0.0311	0.064	*LINC01692*	DemvsCtrl	All CNVs
16093	3	0	2	0	0.0311	0.064	*LINC02573*	DemvsCtrl	All CNVs
16093	1	0	3	0	0.0249	0.0512	*LINC02669*	DemvsCtrl	All CNVs
16093	3	0	1	0	0.0249	0.0512	*MIER2*	DemvsCtrl	All CNVs
16093	3	0	2	0	0.0311	0.064	*MIR548X*	DemvsCtrl	All CNVs
16093	2	0	3	0	0.0311	0.064	*OBSCN-AS1*	DemvsCtrl	All CNVs
16093	1	0	3	0	0.0249	0.0512	*PMS2*	DemvsCtrl	All CNVs
16093	2	0	3	0	0.0311	0.064	*PSMG1*	DemvsCtrl	All CNVs
16093	3	0	1	0	0.0249	0.0512	*SIM2*	DemvsCtrl	All CNVs
16093	3	0	1	0	0.0249	0.0512	*TCERG1L-AS1*	DemvsCtrl	All CNVs
16093	3	0	1	0	0.0249	0.0512	*TMPRSS15*	DemvsCtrl	All CNVs
16093	3	0	1	0	0.0249	0.0512	*UPK1A*	DemvsCtrl	All CNVs
16093	5	0	1	0	0.0373	0.0767	*AMER3*	DemvsCtrl	Duplications
16093	4	0	4	2	0.0621	0.1023	*ANXA11*	DemvsCtrl	Duplications
16093	4	0	1	1	0.0373	0.064	*ARHGEF4*	DemvsCtrl	Duplications
16093	4	0	0	1	0.0311	0.0512	*BCL2L2-PABPN1*	DemvsCtrl	Duplications
16093	5	0	3	2	0.0621	0.1023	*BMS1P21*	DemvsCtrl	Duplications
16093	4	0	0	0	0.0249	0.0512	*CCDC50*	DemvsCtrl	Duplications
16093	8	0	0	0	0.0497	0.1023	*CMTM5*	DemvsCtrl	Duplications
16093	4	0	1	0	0.0311	0.064	*CTNND2*	DemvsCtrl	Duplications
16093	5	0	2	0	0.0435	0.0895	*CXADR*	DemvsCtrl	Duplications
16093	8	0	0	0	0.0497	0.1023	*EFS*	DemvsCtrl	Duplications
16093	4	0	0	1	0.0311	0.0512	*FAM168B*	DemvsCtrl	Duplications
16093	4	0	0	2	0.0373	0.0512	*FKBP6*	DemvsCtrl	Duplications
16093	5	0	0	0	0.0311	0.064	*FRG2DP*	DemvsCtrl	Duplications
16093	4	0	0	0	0.0249	0.0512	*GIMAP6*	DemvsCtrl	Duplications
16093	5	0	1	0	0.0373	0.0767	*GPR148*	DemvsCtrl	Duplications
16093	4	0	2	0	0.0373	0.0767	*GRM7*	DemvsCtrl	Duplications
16093	8	0	0	0	0.0497	0.1023	*IL25*	DemvsCtrl	Duplications
16093	4	0	1	0	0.0311	0.064	*KMT2B*	DemvsCtrl	Duplications
16093	4	0	4	2	0.0621	0.1023	*LINC00857*	DemvsCtrl	Duplications
16093	5	0	0	0	0.0311	0.064	*LINC01115*	DemvsCtrl	Duplications
16093	5	0	0	0	0.0311	0.064	*LINC01566*	DemvsCtrl	Duplications
16093	4	0	0	0	0.0249	0.0512	*LINC01939*	DemvsCtrl	Duplications
16093	7	0	1	0	0.0497	0.1023	*LINC02301*	DemvsCtrl	Duplications
16093	4	0	4	8	0.0994	0.1023	*LOC100130370*	DemvsCtrl	Duplications
16093	6	0	0	0	0.0373	0.0767	*LOC105369306*	DemvsCtrl	Duplications
16093	4	0	0	0	0.0249	0.0512	*LRIG2*	DemvsCtrl	Duplications
16093	4	0	3	2	0.0559	0.0895	*MBL1P*	DemvsCtrl	Duplications
16093	8	0	0	0	0.0497	0.1023	*MHRT*	DemvsCtrl	Duplications
16093	8	0	0	0	0.0497	0.1023	*MIR208A*	DemvsCtrl	Duplications
16093	8	0	0	0	0.0497	0.1023	*MIR208B*	DemvsCtrl	Duplications
16093	4	0	0	0	0.0249	0.0512	*MIR9901*	DemvsCtrl	Duplications
16093	8	0	0	0	0.0497	0.1023	*MYH6*	DemvsCtrl	Duplications
16093	8	0	0	0	0.0497	0.1023	*MYH7*	DemvsCtrl	Duplications
16093	4	0	3	5	0.0746	0.0895	*NCAM2*	DemvsCtrl	Duplications
16093	4	0	0	0	0.0249	0.0512	*OR2L3*	DemvsCtrl	Duplications
16093	4	0	0	0	0.0249	0.0512	*OR4S1*	DemvsCtrl	Duplications
16093	4	0	0	0	0.0249	0.0512	*OR4X1*	DemvsCtrl	Duplications
16093	4	0	0	1	0.0311	0.0512	*PABPN1*	DemvsCtrl	Duplications
16093	5	0	0	0	0.0311	0.064	*PFKP*	DemvsCtrl	Duplications
16093	4	0	0	0	0.0249	0.0512	*PITRM1*	DemvsCtrl	Duplications
16093	4	0	0	0	0.0249	0.0512	*PITRM1-AS1*	DemvsCtrl	Duplications
16093	4	0	4	2	0.0621	0.1023	*PLAC9*	DemvsCtrl	Duplications
16093	4	0	0	0	0.0249	0.0512	*SBK2*	DemvsCtrl	Duplications
16093	4	0	0	0	0.0249	0.0512	*SBK3*	DemvsCtrl	Duplications
16093	5	0	3	2	0.0621	0.1023	*SFTPD*	DemvsCtrl	Duplications
16093	4	0	0	0	0.0249	0.0512	*SLC22A17*	DemvsCtrl	Duplications
16093	4	0	0	0	0.0249	0.0512	*SNORD142*	DemvsCtrl	Duplications
16093	4	0	0	0	0.0249	0.0512	*SNTG2-AS1*	DemvsCtrl	Duplications
16093	4	0	0	0	0.0249	0.0512	*SSC5D*	DemvsCtrl	Duplications
16093	4	0	4	2	0.0621	0.1023	*TMEM254*	DemvsCtrl	Duplications
16093	4	0	4	2	0.0621	0.1023	*TMEM254-AS1*	DemvsCtrl	Duplications
16093	5	0	0	0	0.0311	0.064	*TP53TG3HP*	DemvsCtrl	Duplications
16093	5	0	1	0	0.0373	0.0767	*UTS2B*	DemvsCtrl	Duplications
16093	4	0	1	0	0.0311	0.064	*ZBTB32*	DemvsCtrl	Duplications
16093	0	0	8	0	0.0497	0.1023	*ACMSD*	DemvsCtrl	Deletions
16093	0	1	4	0	0.0311	0.0512	*ARHGEF38*	DemvsCtrl	Deletions
16093	0	1	4	0	0.0311	0.0512	*ARHGEF38-IT1*	DemvsCtrl	Deletions
16093	0	0	8	0	0.0497	0.1023	*CCNT2*	DemvsCtrl	Deletions
16093	0	0	8	0	0.0497	0.1023	*CCNT2-AS1*	DemvsCtrl	Deletions
16093	3	2	5	0	0.0621	0.1023	*COL28A1*	DemvsCtrl	Deletions
16093	0	0	5	0	0.0311	0.064	*FYN*	DemvsCtrl	Deletions
16093	0	1	4	0	0.0311	0.0512	*GNPDA1*	DemvsCtrl	Deletions
16093	0	0	4	0	0.0249	0.0512	*GSTCD*	DemvsCtrl	Deletions
16093	7	14	4	0	0.1553	0.1407	*HSF4*	DemvsCtrl	Deletions
16093	2	1	4	0	0.0435	0.0767	*LINC02679*	DemvsCtrl	Deletions
16093	0	1	5	0	0.0373	0.064	*MIR599*	DemvsCtrl	Deletions
16093	0	1	5	0	0.0373	0.064	*MIR875*	DemvsCtrl	Deletions
16093	1	0	4	0	0.0311	0.064	*PCDH17*	DemvsCtrl	Deletions
16093	2	1	4	0	0.0435	0.0767	*SFTPA1*	DemvsCtrl	Deletions
16093	2	1	4	0	0.0435	0.0767	*SFTPA2*	DemvsCtrl	Deletions
16093	0	0	6	0	0.0373	0.0767	*TET2*	DemvsCtrl	Deletions
16093	0	0	6	0	0.0373	0.0767	*TET2-AS1*	DemvsCtrl	Deletions
16093	1	1	4	0	0.0373	0.064	*TMEM114*	DemvsCtrl	Deletions
16093	4	2	5	0	0.0684	0.1151	*VAT1L*	DemvsCtrl	Deletions
16093	1	1	9	0	0.0684	0.1279	*VPS13B*	DemvsCtrl	Deletions
16093	5	7	5	0	0.1056	0.1279	*ZDHHC11B*	DemvsCtrl	Deletions
16093	0	0	5	0	0.0311	0.064	*ZNF681*	DemvsCtrl	Deletions

Supplementary Table 5. Pathway enrichment analysis of genes affected by CNVs exclusive to dementia cases.

This table presents the results of pathway enrichment analysis (KEGG and Gene Ontology) performed on genes affected by CNVs observed exclusively in dementia cases. Enriched pathways may highlight biological processes and molecular mechanisms potentially involved in dementia pathogenesis.

Enrichment	CNVs	Category	Subcategory	ID	Description	GeneRatio	BgRatio	RichFactor	FoldEnrichment	zScore	pvalue	p.adjust	qvalue	geneID	Count	Gene Names
KEGG	Deletions	Human Diseases	Infectious disease: bacterial	hsa05133	Pertussis	2/8	78/8546	0.026	27.391	7.167	2.22E-03	4.89E-02	4.21E-02	653509/729238	2	*SFTPA1, SFTPA2*
KEGG	Duplications	Human Diseases	Cardiovascular disease	hsa05416	Viral myocarditis	3/19	70/8546	0.043	19.277	7.247	4.64E-04	1.76E-02	1.42E-02	1525/4624/4625	3	*CXADR, MYH6, MYH7*
KEGG	Duplications	Organismal Systems	Endocrine system	hsa04919	Thyroid hormone signaling pathway	3/19	122/8546	0.025	11.060	5.283	2.33E-03	4.42E-02	3.55E-02	4624/4625/5214	3	*MYH6, MYH7, PFKP*
Gene Ontology	Duplications	NA	NA	GO:0014888	striated muscle adaptation	4/33	57/18986	0.070	40.374	12.423	2.80E-06	1.37E-03	9.69E-04	406990/100126336/4624/4625	4	*MIR208A, MIR208B, MYH6, MYH7*
Gene Ontology	Duplications	NA	NA	GO:0003299	muscle hypertrophy in response to stress	3/33	32/18986	0.094	53.938	12.506	2.29E-05	2.81E-03	1.98E-03	406990/4624/4625	3	*MIR208A, MYH6, MYH7*
Gene Ontology	Duplications	NA	NA	GO:0014887	cardiac muscle adaptation	3/33	32/18986	0.094	53.938	12.506	2.29E-05	2.81E-03	1.98E-03	406990/4624/4625	3	*MIR208A, MYH6, MYH7*
Gene Ontology	Duplications	NA	NA	GO:0014898	cardiac muscle hypertrophy in response to stress	3/33	32/18986	0.094	53.938	12.506	2.29E-05	2.81E-03	1.98E-03	406990/4624/4625	3	*MIR208A, MYH6, MYH7*
Gene Ontology	Duplications	NA	NA	GO:0043500	muscle adaptation	4/33	122/18986	0.033	18.863	8.259	5.75E-05	5.65E-03	3.98E-03	406990/100126336/4624/4625	4	*MIR208A, MIR208B, MYH6, MYH7*
Gene Ontology	Duplications	NA	NA	GO:0086016	AV node cell action potential	2/33	11/18986	0.182	104.606	14.342	1.60E-04	1.08E-02	7.59E-03	1525/406990	2	*CXADR, MIR208A*

Supplementary Table 6. Duplications identified in the *14q11.2* region.
This table lists the CNV duplications effecting the IL25 gene region (14q11.2), based on human genome build hg19.

nIndividual	Pheno	Age	Type	Chr	Start	End	nSNPs	Length	Target_gene	Annotated_Genes
1	AD	79	Duplication	14	23766591	23905600	1190	139010	IL25	*BCL2L2,BCL2L2-PABPN1,CMTM5,EFS,IL25,MHRT,MIR208A,MIR208B,MYH6,MYH7,PABPN1,PPP1R3E,SLC22A17*
2	AD	83	Duplication	14	23777099	23907608	1188	130510	IL25	*BCL2L2,BCL2L2-PABPN1,CMTM5,EFS,IL25,MHRT,MIR208A,MIR208B,MYH6,MYH7,PABPN1,SLC22A17*
3	AD	81	Duplication	14	23777099	23923165	1193	146067	IL25	*BCL2L2,BCL2L2-PABPN1,CMTM5,EFS,IL25,MHRT,MIR208A,MIR208B,MYH6,MYH7,PABPN1,SLC22A17*
4	AD	72	Duplication	14	23789955	23900093	1015	110139	IL25	*BCL2L2-PABPN1,CMTM5,EFS,IL25,MHRT,MIR208A,MIR208B,MYH6,MYH7,PABPN1,SLC22A17*
5	AD	85	Duplication	14	23822800	23910018	1175	87219	IL25	*CMTM5,EFS,IL25,MHRT,MIR208A,MIR208B,MYH6,MYH7*
6	AD	62	Duplication	14	23828893	23907608	1165	78716	IL25	*CMTM5,EFS,IL25,MHRT,MIR208A,MIR208B,MYH6,MYH7*
7	AD	73	Duplication	14	23828893	23907608	1165	78716	IL25	*CMTM5,EFS,IL25,MHRT,MIR208A,MIR208B,MYH6,MYH7*
8	AD	75	Duplication	14	23828893	23910018	1166	81126	IL25	*CMTM5,EFS,IL25,MHRT,MIR208A,MIR208B,MYH6,MYH7*

Supplementary Table 7. ClinVar CNVs overlapping the *VPS13B* region identified in our cohort.

This table displays CNVs from the ClinVar database that overlap with the genomic region affected in individuals from our study cohort carrying CNVs in the VPS13B gene. The individuals are labeled as Indiv1-9 across the columns. For each individual, the values range from 0 to 1, indicating whether the ClinVar CNV region overlaps with the CNV identified in our cohort (0 = no overlap, 1 = overlap).
The table also includes information on clinical significance, phenotype associations, and genomic coordinates.

Name	Gene(s)	Conditions	Clinical significance Last reviewed	Clinical significance r	Type	Review status	Accession	GRCh37Chr	GRCh37Location	GRCh38Loc	GRCh38Loc	VariationID	AlleleIDs	Indiv1	Indiv2	Indiv3	Indiv4	Indiv5	Indiv6	Indiv7	Indiv8	Indiv9
GRCh37/hg19 8q22.2(chr8:100205147-100252754)x3	*VPS13B*	not provided	Benign(Last reviewed: Aug 2, 2015)	Benign	dup	no assertion criteria provided	VCV000611100	8	100205147 - 100252754			611100	602436	0	0	0	0	0	0	1	0	0
GRCh37/hg19 8q22.2(chr8:100032844-100528645)x3	*VPS13B*	not provided	Benign(Last reviewed: Aug 28, 2013)	Benign	dup	no assertion criteria provided	VCV000611096	8	100032844 - 100528645			611096	602432	1	1	1	1	1	1	1	1	1
GRCh37/hg19 8q22.2(chr8:100242018-100515757)x1	*VPS13B*	not provided	Benign(Last reviewed: Dec 14, 2016)	Benign	del	no assertion criteria provided	VCV000611101	8	100242018 - 100515757	99229790	99503529	611101	602437	1	1	1	1	1	1	0	1	1
GRCh37/hg19 8q22.2(chr8:100111153-100544470)x3	*VPS13B*	not provided	Benign(Last reviewed: Jul 16, 2013)	Benign	dup	no assertion criteria provided	VCV000611099	8	100111153 - 100544470			611099	602435	1	1	1	1	1	1	1	1	1
GRCh37/hg19 8q22.2(chr8:100111153-100371704)x3	*VPS13B*	not provided	Benign(Last reviewed: Jul 25, 2013)	Benign	dup	no assertion criteria provided	VCV000611098	8	100111153 - 100371704			611098	602434	0	0	0	0	0	0	1	0	1
GRCh37/hg19 8q22.2(chr8:100111153-100351288)x3	*VPS13B*	not provided	Benign(Last reviewed: Sep 3, 2013)	Benign	dup	no assertion criteria provided	VCV000611097	8	100111153 - 100351288			611097	602433	0	0	0	0	0	0	1	0	1
GRCh37/hg19 8q22.2(chr8:100342240-100494359)x1	*VPS13B*	not provided	Likely benign(Last reviewed: Jan 10, 2020)	Likely_Benign	del	no assertion criteria provided	VCV000979845	8	100342240 - 100494359	99330012	99482131	979845	967923	1	1	1	1	1	1	0	1	1
GRCh38/hg38 8q22.2(chr8:99394612-99622937)x1	*MIR599|MIR875|VPS1*	See cases	Likely benign(Last reviewed: Oct 21, 2014)	Likely_Benign	del	no assertion criteria provided	VCV000153352	8	100406840 - 100635165	99394612	99622937	153352	163103	1	1	1	0	1	1	0	1	0
GRCh37/hg19 8q22.2(chr8:100324335-100564600)x1	*VPS13B*	not provided	Likely benign(Last reviewed: Oct 29, 2019)	Likely_Benign	del	no assertion criteria provided	VCV000979844	8	100324335 - 100564600	99312107	99552372	979844	967922	1	1	1	1	1	1	0	1	1
NC_000008.11:g.(?_99134631)_(99511512_?)dup	*VPS13B*	Cohen syndrome	Likely pathogenic(Last reviewed: Dec 24, 2019)	Likely_pathogenic	dup	criteria provided, single submitter	VCV000831712	8	100146859 - 100523740			831712	820012	1	1	1	1	1	1	1	1	1
NC_000008.10:g.(?_100396426)_(100479872_?)dup	*VPS13B*	Cohen syndrome	Likely pathogenic(Last reviewed: Jun 10, 2020)	Likely_pathogenic	dup	criteria provided, single submitter	VCV001066497	8	100396426 - 100479872			1066497	1054435	1	1	1	0	1	1	0	1	0
NC_000008.11:g.(?_99209582)_(99275264_?)del	*VPS13B*	Cohen syndrome	Likely pathogenic(Last reviewed: May 29, 2019)	Likely_pathogenic	del	criteria provided, single submitter	VCV000832260	8	100221810 - 100287492	99209582	99275264	832260	820018	0	0	0	0	0	0	1	0	0
NC_000008.10:g.(?_100026017)_(100887894_?)del	*VPS13B*	Cohen syndrome	Pathogenic(Last reviewed: Apr 12, 2021)	Pathogenic	del	criteria provided, single submitter	VCV001454059	8	100026017 - 100887894	99013789	99875666	1454059	1366667	1	1	1	1	1	1	1	1	1
GRCh37/hg19 8q22.2(chr8:100168626-100298366)	*VPS13B*	Cohen syndrome	Pathogenic(Last reviewed: Apr 16, 2020)	Pathogenic	del	no assertion criteria provided	VCV000915968	8	100168626 - 100298366	99156398	99286138	915968	904193	0	0	0	0	0	0	1	0	0
GRCh37/hg19 8q22.2(chr8:100232749-100493790)	*VPS13B*	Cohen syndrome	Pathogenic(Last reviewed: Apr 16, 2020)	Pathogenic	del	no assertion criteria provided	VCV000915973	8	100232749 - 100493790	99220521	99481562	915973	904198	1	1	1	1	1	1	0	1	1
GRCh37/hg19 8q22.2(chr8:100274176-100477297)	*VPS13B*	Cohen syndrome	Pathogenic(Last reviewed: Apr 16, 2020)	Pathogenic	del	no assertion criteria provided	VCV000915972	8	100274176 - 100477297	99261948	99465069	915972	904197	0	1	1	1	1	1	0	1	1
NC_000008.10:g.(?_100108530)_(100589871_?)del	*VPS13B*	Cohen syndrome	Pathogenic(Last reviewed: Aug 12, 2021)	Pathogenic	del	criteria provided, single submitter	VCV001456598	8	100108530 - 100589871	99096302	99577643	1456598	1454700	1	1	1	1	1	1	1	1	1
NC_000008.10:g.(?_100182257)_(100479872_?)del	*VPS13B*	Cohen syndrome	Pathogenic(Last reviewed: Aug 12, 2021)	Pathogenic	del	criteria provided, single submitter	VCV001070729	8	100182257 - 100479872	99170029	99467644	1070729	1057318	1	1	1	1	1	1	1	1	1
NC_000008.10:g.(?_100205084)_(100454883_?)del	*VPS13B*	Cohen syndrome	Pathogenic(Last reviewed: Aug 12, 2021)	Pathogenic	del	criteria provided, single submitter	VCV001451479	8	100205084 - 100454883	99192856	99442655	1451479	1425881	0	1	0	1	1	0	1	1	1
NC_000008.10:g.(?_100286406)_(100874194_?)del	*VPS13B*	Cohen syndrome	Pathogenic(Last reviewed: Aug 12, 2021)	Pathogenic	del	criteria provided, single submitter	VCV001070731	8	100286406 - 100874194	99274178	99861966	1070731	1057320	1	1	1	1	1	1	0	1	1
NC_000008.10:g.(?_100286416)_(100568891_?)del	*VPS13B*	Cohen syndrome	Pathogenic(Last reviewed: Aug 12, 2021)	Pathogenic	del	criteria provided, single submitter	VCV001460182	8	100286416 - 100568891	99274188	99556663	1460182	1481538	1	1	1	1	1	1	0	1	1
NC_000008.10:g.(?_100396426)_(100523750_?)del	*VPS13B*	Cohen syndrome	Pathogenic(Last reviewed: Aug 12, 2021)	Pathogenic	del	criteria provided, single submitter	VCV001070733	8	100396426 - 100523750	99384198	99511522	1070733	1057322	1	1	1	0	1	1	0	1	0
NC_000008.10:g.(?_100403775)_(100568891_?)del	*VPS13B*	Cohen syndrome	Pathogenic(Last reviewed: Aug 12, 2021)	Pathogenic	del	criteria provided, single submitter	VCV001458204	8	100403775 - 100568891	99391547	99556663	1458204	1467919	1	1	1	0	1	1	0	1	0
NC_000008.11:g.(?_99391557)_(99556653_?)del	*VPS13B*	Cohen syndrome	Pathogenic(Last reviewed: Aug 19, 2019)	Pathogenic	del	criteria provided, single submitter	VCV000831437	8	100403785 - 100568881	99391557	99556653	831437	820019	1	1	1	0	1	1	0	1	0
NC_000008.11:g.(?_99384188)_(99721067_?)del	*VPS13B|LOC1101208*	Cohen syndrome	Pathogenic(Last reviewed: Aug 31, 2021)	Pathogenic	del	criteria provided, single submitter	VCV000664445	8	100396416 - 100733295	99384188	99721067	664445	651878	1	1	1	0	1	1	0	1	0
NC_000008.10:g.(?_100146850)_(100287492_?)del	*VPS13B*	Cohen syndrome	Pathogenic(Last reviewed: Aug 4, 2021)	Pathogenic	del	criteria provided, single submitter	VCV001074933	8	100146850 - 100287492	99134622	99275264	1074933	1057314	0	0	0	0	0	0	1	0	0
NC_000008.11:g.(?_99096302)_(99556663_?)del	*VPS13B*	Cohen syndrome	Pathogenic(Last reviewed: Aug 9, 2019)	Pathogenic	del	criteria provided, single submitter	VCV000830631	8	100108530 - 100568891	99096302	99556663	830631	820006	1	1	1	1	1	1	1	1	1
NC_000008.11:g.(?_99391537)_(99521030_?)del	*VPS13B*	Cohen syndrome	Pathogenic(Last reviewed: Dec 14, 2018)	Pathogenic	del	criteria provided, single submitter	VCV000650184	8	100403765 - 100533258	99391537	99521030	650184	651730	1	1	1	0	1	1	0	1	0
NC_000008.11:g.(?_99209572)_(99391724_?)del	*VPS13B*	Cohen syndrome	Pathogenic(Last reviewed: Dec 5, 2017)	Pathogenic	del	criteria provided, single submitter	VCV000528872	8	100221800 - 100403952	99209572	99391724	528872	523181	0	0	0	1	0	0	1	0	1
NC_000008.10:g.(?_100454619)_(100533248_?)del	*VPS13B*	Cohen syndrome	Pathogenic(Last reviewed: Dec 7, 2021)	Pathogenic	del	criteria provided, single submitter	VCV001076760	8	100454619 - 100533248	99442391	99521020	1076760	1057324	1	0	1	0	0	1	0	0	0
NC_000008.11:g.(?_99121167)_(99721057_?)del	*VPS13B*	Cohen syndrome	Pathogenic(Last reviewed: Feb 11, 2019)	Pathogenic	del	criteria provided, single submitter	VCV000833226	8	100133395 - 100733285	99121167	99721057	833226	820011	1	1	1	1	1	1	1	1	1
NC_000008.11:g.(?_99431517)_(99721067_?)del	*LOC110120801|MIR5*	Cohen syndrome	Pathogenic(Last reviewed: Feb 16, 2018)	Pathogenic	del	criteria provided, single submitter	VCV000584084	8	100443745 - 100733295	99431517	99721067	584084	564568	1	0	1	0	0	1	0	0	0
NC_000008.10:g.(?_100221800)_(100589881_?)del	*VPS13B*	Cohen syndrome	Pathogenic(Last reviewed: Feb 20, 2020)	Pathogenic	del	criteria provided, single submitter	VCV001070730	8	100221800 - 100589881	99209572	99577653	1070730	1057319	1	1	1	1	1	1	1	1	1
NC_000008.11:g.(?_99274178)_(99721067_?)del	*LOC110120801|MIR5*	Cohen syndrome	Pathogenic(Last reviewed: Jan 3, 2018)	Pathogenic	del	criteria provided, single submitter	VCV000528873	8	100286406 - 100733295	99274178	99721067	528873	523529	1	1	1	1	1	1	0	1	1
NC_000008.11:g.(?_99384188)_(99511532_?)del	*VPS13B*	Cohen syndrome	Pathogenic(Last reviewed: Jul 13, 2018)	Pathogenic	del	criteria provided, single submitter	VCV000660699	8	100396416 - 100523760	99384188	99511532	660699	651830	1	1	1	0	1	1	0	1	0
NC_000008.10:g.100246250_100460500del	*VPS13B*	Cohen syndrome	Pathogenic(Last reviewed: Mar 30, 2021)	Pathogenic	del	criteria provided, single submitter	VCV001048632	8	100246250 - 100460500	99234022	99448272	1048632	1036719	0	1	0	1	1	0	0	1	1
NC_000008.11:g.(?_99096292)_(99577653_?)del	*MIR599|MIR875|VPS1*	Cohen syndrome	Pathogenic(Last reviewed: May 18, 2018)	Pathogenic	del	criteria provided, single submitter	VCV000584321	8	100108520 - 100589881	99096292	99577653	584321	564561	1	1	1	1	1	1	1	1	1
NC_000008.10:g.(?_100108530)_(100287492_?)del	*VPS13B*	Cohen syndrome	Pathogenic(Last reviewed: Nov 16, 2021)	Pathogenic	del	criteria provided, single submitter	VCV001070870	8	100108530 - 100287492	99096302	99275264	1070870	1057310	0	0	0	0	0	0	1	0	0
NC_000008.10:g.(?_100205094)_(100287492_?)del	*VPS13B*	Cohen syndrome	Pathogenic(Last reviewed: Nov 9, 2021)	Pathogenic	del	criteria provided, single submitter	VCV001456587	8	100205094 - 100287492	99192866	99275264	1456587	1454689	0	0	0	0	0	0	1	0	0
NC_000008.10:g.(?_100443745)_(100589881_?)del	*VPS13B*	Cohen syndrome	Pathogenic(Last reviewed: Oct 10, 2021)	Pathogenic	del	criteria provided, single submitter	VCV001076759	8	100443745 - 100589881	99431517	99577653	1076759	1057323	1	0	1	0	0	1	0	0	0
GRCh37/hg19 8q22.2(chr8:100120427-100423020)x1	*VPS13B*	not provided	Pathogenic(Last reviewed: Sep 15, 2017)	Pathogenic	del	no assertion criteria provided	VCV000689112	8	100120427 - 100423020	99108199	99410792	689112	676704	0	0	0	1	0	0	1	1	1
NC_000008.10:g.(?_100443755)_(100568891_?)del	*VPS13B*	Cohen syndrome	Pathogenic(Last reviewed: Sep 19, 2021)	Pathogenic	del	criteria provided, single submitter	VCV001457471	8	100443755 - 100568891	99431527	99556663	1457471	1393412	1	0	1	0	0	1	0	0	0
NC_000008.11:g.(?_99274178)_(99577653_?)del	*MIR599|MIR875|VPS1*	Cohen syndrome	Pathogenic(Last reviewed: Sep 22, 2018)	Pathogenic	del	criteria provided, single submitter	VCV000657371	8	100286406 - 100589881	99274178	99577653	657371	651768	1	1	1	1	1	1	0	1	1
NC_000008.10:g.(?_100286416)_(100454873_?)del	*VPS13B*	Cohen syndrome	Pathogenic(Last reviewed: Sep 29, 2021)	Pathogenic	del	criteria provided, single submitter	VCV001454066	8	100286416 - 100454873	99274188	99442645	1454066	1366674	0	1	0	1	1	0	0	1	1
GRCh38/hg38 8q22.2(chr8:99278660-99451719)x1	*VPS13B*	See cases	Uncertain significance(Last reviewed: Aug 12, 2011)	Uncertain	del	criteria provided, single submitter	VCV000057435	8	100290888 - 100463947	99278660	99451719	57435	72030	0	1	0	1	1	0	0	1	1
NC_000008.11:g.(?_99013769)_(99875686_?)dup	*VPS13B*	Cohen syndrome	Uncertain significance(Last reviewed: Dec 27, 2020)	Uncertain	dup	criteria provided, single submitter	VCV000833046	8	100025997 - 100887914			833046	820004	1	1	1	1	1	1	1	1	1
GRCh37/hg19 8q22.2(chr8:100276485-100494359)x1	*VPS13B*	not provided	Uncertain significance(Last reviewed: Dec 4, 2018)	Uncertain	del	no assertion criteria provided	VCV000815148	8	100276485 - 100494359	99264257	99482131	815148	803357	1	1	1	1	1	1	0	1	1
GRCh37/hg19 8q22.2(chr8:100251329-100422704)x1	*VPS13B*	not provided	Uncertain significance(Last reviewed: Feb 1, 2021)	Uncertain	del	no assertion criteria provided	VCV001341198	8	100251329 - 100422704	99239101	99410476	1341198	1332406	0	0	0	1	0	0	0	1	1
GRCh37/hg19 8q22.2(chr8:100121961-100331982)x1	*VPS13B*	not provided	Uncertain significance(Last reviewed: Mar 15, 2019)	Uncertain	del	no assertion criteria provided	VCV000563456	8	100121961 - 100331982	99109733	99319754	563456	554640	0	0	0	0	0	0	1	0	0
GRCh38/hg38 8q22.2(chr8:99055243-99917250)x1	*COX6C|LOC1101208*	See cases	Uncertain significance(Last reviewed: Mar 18, 2014)	Uncertain	del	no assertion criteria provided	VCV000154273	8	100067471 - 100929478	99055243	99917250	154273	164024	1	1	1	1	1	1	1	1	1
GRCh37/hg19 8q22.2(chr8:100356422-100777020)x1	*VPS13B*	not provided	Uncertain significance(Last reviewed: Sep 12, 2018)	Uncertain	del	no assertion criteria provided	VCV000686925	8	100356422 - 100777020	99344194	99764792	686925	674517	1	1	1	1	1	1	0	1	0

Supplementary Table 8. Counts of CNVs affecting Alzheimer's Disease-related genes.

This table summarizes the number of deletions and duplications that overlap with known Alzheimer's disease-related genes (based in Bellenguez et al. 2022) in our study cohort. The table includes gene names, CNV type and the count of affected individuals for each gene. Alzheimer's disease cases (AD)=7,818, Healthy controls (Ctrl) = 8,275.

	Deletions			Duplications		
Gene name	Total	AD	Ctrl	Total	AD	Ctrl
ABCA1	1	1	0	1	1	0
ABCA7	15	11	4			
ABI3	1	1	0			
ACE	3	1	2			
ADAM17				1	1	0
ADAMTS1				2	2	0
ANK3				1	1	0
ANKH				1	0	1
APP	2	2	0	1	1	0
BIN1	1	0	1			
BLNK				1	0	1
CLNK				4	2	2
CLU				1	0	1
COX7C	1	0	1			
CR1	1	0	1	3	1	2
CTSB				1	0	1
DOC2A	2	1	1	7	5	2
EED				1	1	0
FOXF1	3	0	3			
GRN	1	1	0			
HLA-DQA1	1	1	0			
ICA1	1	1	0	10	4	6
IDUA				10	6	4
INPP5D	1	1	0			
KLF16	25	9	16			
LILRB2				18	6	12
MAPT	14	1	13			
MS4A4A				1	0	1
MYO15A	1	0	1			
NCK2				1	0	1
PLCG2	2	0	2	1	1	0
PTK2B	4	0	4	1	0	1
RASGEF1C				2	1	1
RBCK1	1	0	1			
SCIMP	1	0	1			
SHARPIN	22	10	12	35	17	18
SIGLEC11	1	1	0	2	0	2
SLC24A4	1	0	1			
SLC2A4RG	9	4	5	7	3	4
SPPL2A				2	0	2
TMEM106B	1	0	1	2	0	2
TNIP1	2	2	0			
TPCN1	1	0	1	3	1	2
UMAD1	2	2	0	1	1	0
WDR12	1	1	0			
TOTAL	122	51	71	123	57	66

Supplementary Table 9. CNV information for *MAPT*, *ABCA7* and *APP* Genes.

This table provides detailed information on copy number variants (CNVs), including deletions and duplications, identified in these key loci previously implicated in Alzheimer's disease (*MAPT*, *ABCA7* and *APP*). Genomic positions based on human genome build hg19.

nIndividual	Pheno	Age	Type	Chr	Start	End	nSNPs	Length	Target_gene	Annotated_Genes
1	AD	84.15	Deletion	17	44076665	44566824	177	490160	MAPT	*ARL17A,ARL17B,KANSL1,KANSL1-AS1,LRRC37A,MAPT,NSFP1,STH*
2	Controls	52	Deletion	17	43901558	44111613	239	210056	MAPT	*CRHR1,KANSL1,LINC02210-CRHR1,MAPT,MAPT-AS1,MAPT-IT1,SPPL2C,STH*
3	Controls	78	Deletion	17	44071294	44358286	185	286993	MAPT	*ARL17B,KANSL1,KANSL1-AS1,MAPT,STH*
4	Controls	77	Deletion	17	44017124	44118848	128	101725	MAPT	*KANSL1,MAPT,STH*
5	Controls	74	Deletion	17	43901558	44081268	204	179711	MAPT	*CRHR1,LINC02210-CRHR1,MAPT,MAPT-AS1,MAPT-IT1,SPPL2C,STH*
6	Controls	38	Deletion	17	43922357	44111613	202	189257	MAPT	*KANSL1,MAPT,MAPT-AS1,MAPT-IT1,SPPL2C,STH*
7	Controls	33	Deletion	17	43912342	44358286	346	445945	MAPT	*ARL17B,CRHR1,KANSL1,KANSL1-AS1,LINC02210-CRHR1,MAPT,MAPT-AS1,MAPT-IT1,SPPL2C,STH*
8	Controls	19	Deletion	17	44038785	44111613	105	72829	MAPT	*KANSL1,MAPT,STH*
9	Controls	24	Deletion	17	43901558	44118848	248	217291	MAPT	*CRHR1,KANSL1,LINC02210-CRHR1,MAPT,MAPT-AS1,MAPT-IT1,SPPL2C,STH*
10	Controls	66	Deletion	17	43972581	44118848	161	146268	MAPT	*KANSL1,MAPT,MAPT-AS1,MAPT-IT1,STH*
11	Controls	81	Deletion	17	44071294	44358286	185	286993	MAPT	*ARL17B,KANSL1,KANSL1-AS1,MAPT,STH*
12	Controls	74	Deletion	17	44078618	44169581	74	90964	MAPT	*KANSL1,MAPT*
13	Controls	44	Deletion	17	44071294	44358286	185	286993	MAPT	*ARL17B,KANSL1,KANSL1-AS1,MAPT,STH*
14	Controls	59	Deletion	17	44054696	44169581	124	114886	MAPT	*KANSL1,MAPT,STH*
15	AD	87.39	Deletion	19	998855	1120329	228	121475	ABCA7	*ABCA7,ARHGAP45,CNN2,GRIN3B,POLR2E,RNU6-1,RNU6-2,RNU6-7,RNU6-8,RNU6-9,TMEM259*
16	AD	61	Deletion	19	580710	1999338	1191	1418629	ABCA7	*ABCA7,ABHD17A,ADAT3,ARHGAP45,ARID3A,ATP8B3,AZU1,BSG,BTBD2,CFD,CNN2,CSNK1G2,CSNK1G2-AS1,ELANE,FSTL3,GRIN3B,KISS1R,KLF16,LOC100288123,MBD3,MED16,MEX3D,MIR1909,MIR3187,MIR4745,MISP,ONECUT3,PALM,PLK5,PLPPR3,POLR2E,PRSS57,PRTN3,PTBP1,R3HDM4,REXO1,RNU6-1,RNU6-2,RNU6-7,RNU6-8,RNU6-9,SCAMP4,TCF3,TMEM259,UQCR11,WDR18*
17	AD	79.87	Deletion	19	1063443	1156457	96	93015	ABCA7	*ABCA7,ARHGAP45,POLR2E,*
18	AD	71.54	Deletion	19	982698	1055191	145	72494	ABCA7	*ABCA7,CNN2,GRIN3B,RNU6-1,RNU6-2,RNU6-7,RNU6-8,RNU6-9,TMEM259,WDR18*
19	AD	71.58	Deletion	19	1030139	1090803	143	60665	ABCA7	*ABCA7,ARHGAP45,CNN2,POLR2E*
20	Controls	65	Deletion	19	1003094	1090803	196	87710	ABCA7	*ABCA7,ARHGAP45,CNN2,GRIN3B,POLR2E,RNU6-1,RNU6-2,RNU6-7,RNU6-8,RNU6-9,TMEM259*
21	Controls	51.08	Deletion	19	1003094	1189482	257	186389	ABCA7	*ABCA7,ARHGAP45,CNN2,GRIN3B,POLR2E,RNU6-1,RNU6-2,RNU6-7,RNU6-8,RNU6-9,TMEM259*
22	AD	80.05	Deletion	19	998855	1090803	198	91949	ABCA7	*ABCA7,ARHGAP45,CNN2,GRIN3B,POLR2E,RNU6-1,RNU6-2,RNU6-7,RNU6-8,RNU6-9,TMEM259*
23	AD	85	Deletion	19	1003094	1054060	120	50967	ABCA7	*ABCA7,CNN2,GRIN3B,RNU6-1,RNU6-2,RNU6-7,RNU6-8,RNU6-9,TMEM259*
24	AD	82.32	Deletion	19	1030139	1090803	143	60665	ABCA7	*ABCA7,ARHGAP45,CNN2,POLR2E*
25	AD	79	Deletion	19	348743	2372678	1690	2023936	ABCA7	*ABCA7,ABHD17A,ADAT3,AMH,ARHGAP45,ARID3A,ATP8B3,AZU1,BSG,BTBD2,C2CD4C,CDC34,CFD,CNN2,CSNK1G2,CSNK1G2-AS1,DOT1L,ELANE,FSTL3,GRIN3B,GZMM,JSRP1,KISS1R,KLF16,LINGO3,LOC100288123,LSM7,MADCAM1,MBD3,MED16,MEX3D,MIR1227,MIR1909,MIR3187,MIR4321,MIR4745,MIR6789,MISP,MKNK2,MOB3A,OAZ1,ODF3L2,ONECUT3,PALM,PEAK3,PLEKHJ1,PLK5,PLPPR3,POLR2E,PRSS57,PRTN3,PTBP1,R3HDM4,REXO1,RNU6-1,RNU6-2,RNU6-7,RNU6-8,RNU6-9,SCAMP4,SF3A2,SHC2,SPPL2B,TCF3,THEG,TMEM259,TPGS1,UQCR11,WDR18*
26	AD	82	Deletion	19	791153	4062220	2229	3271068	ABCA7	*ABCA7,ABHD17A,ADAT3,AMH,APBA3,ARHGAP45,ARID3A,ATCAY,ATP8B3,AZU1,BTBD2,CACTIN-AS1,CELF5,CFD,CNN2,CSNK1G2,CSNK1G2-AS1,DAPK3,DIRAS1,DOHH,DOT1L,EEF2,ELANE,GADD45B,GIPC3,GNA11,GNA15,GNG7,GRIN3B,JSRP1,KISS1R,KLF16,LINC01775,LINGO3,LMNB2,LOC100288123,LOC100996351,LSM7,MATK,MBD3,MED16,MEX3D,MIR1227,MIR1909,MIR3187,MIR4321,MIR4745,MIR637,MIR6789,MIR7108,MIR7850,MKNK2,MOB3A,MRPL54,NCLN,NFIC,NMRK2,OAZ1,ONECUT3,PEAK3,PIAS4,PLEKHJ1,PLK5,PLPPR3,POLR2E,PRTN3,PTBP1,R3HDM4,RAX2,REXO1,RNU6-1,RNU6-2,RNU6-7,RNU6-8,RNU6-9,S1PR4,SCAMP4,SF3A2,SGTA,SLC39A3,SMIM24,SNORD37,SPPL2B,TBXA2R,TCF3,THOP1,TIMM13,TJP3,TLE2,TLE5,TLE6,TMEM259,TMPRSS9,UQCR11,WDR18,ZBTB7A,ZFR2,ZNF554,ZNF555,ZNF556,ZNF57,ZNF77*
27	Controls	38	Deletion	19	1040765	1506135	423	465371	ABCA7	*ABCA7,ARHGAP45,POLR2E,*
28	Controls	67	Deletion	19	375220	4868765	3146	4493546	ABCA7	*ABCA7,ABHD17A,ADAT3,AMH,ANKRD24,APBA3,ARHGAP45,ARID3A,ATCAY,ATP8B3,AZU1,BSG,BTBD2,C2CD4C,CACTIN-AS1,CDC34,CELF5,CFD,CHAF1A,CNN2,CREB3L3,CSNK1G2,CSNK1G2-AS1,DAPK3,DIRAS1,DOHH,DOT1L,DPP9,DPP9-AS1,EBI3,EEF2,ELANE,FEM1A,FSD1,FSTL3,GADD45B,GIPC3,GNA11,GNA15,GNG7,GRIN3B,GZMM,HDGFL2,JSRP1,KISS1R,KLF16,LINC01775,LINGO3,LMNB2,LOC100288123,LOC100996351,LRG1,LSM7,MADCAM1,MAP2K2,MATK,MBD3,MED16,MEX3D,MIR1227,MIR1909,MIR3187,MIR4321,MIR4745,MIR4746,MIR637,MIR6789,MIR7-3,MIR7-3HG,MIR7108,MIR7850,MISP,MKNK2,MOB3A,MPND,MRPL54,MYDGF,NCLN,NFIC,NMRK2,OAZ1,ODF3L2,ONECUT3,PALM,PEAK3,PIAS4,PLEKHJ1,PLIN3,PLIN4,PLIN5,PLK5,PLPPR3,POLR2E,PRSS57,PRTN3,PTBP1,R3HDM4,RAX2,REXO1,RNU6-1,RNU6-2,RNU6-7,RNU6-8,RNU6-9,S1PR4,SCAMP4,SEMA6B,SF3A2,SGTA,SH3GL1,SHC2,SHD,SIRT6,SLC39A3,SMIM24,SNORD37,SPPL2B,STAP2,TBXA2R,TCF3,THEG,THOP1,TICAM1,TIMM13,TJP3,TLE2,TLE5,TLE6,TMEM259,TMIGD2,TMPRSS9,TNFAIP8L1,TPGS1,UBXN6,UQCR11,WDR18,YJU2,ZBTB7A,ZFR2,ZNF554,ZNF555,ZNF556,ZNF57,ZNF77*
29	AD	91	Deletion	19	929186	1134673	299	205488	ABCA7	*ABCA7,ARHGAP45,ARID3A,CNN2,GRIN3B,POLR2E,RNU6-1,RNU6-2,RNU6-7,RNU6-8,RNU6-9,TMEM259,WDR18*
30	AD	69.15	Duplication	21	10873592	27766383	3082	16892792	APP	*ABCC13,ANKRD20A11P,ANKRD30BP2,APP,ATP5PF,BAGE,BAGE2,BAGE3,BAGE4,BAGE5,BTG3,BTG3-AS1,C21orf91,C21orf91-OT1,CHODL,CHODL-AS1,CXADR,CYP4F29P,CYYR1-AS1,D21S2088E,GABPA,HSPA13,JAM2,LINC00158,LINC00308,LINC00317,LINC00320,LINC00515,LINC01425,LINC01549,LINC01674,LINC01683,LINC01684,LINC01687,LINC01689,*[illegible]*,USP25*
31	OtherDem	71.16	Deletion	21	27181935	27265520	27	83586	APP	*APP*
32	OtherDem	63.81	Deletion	21	27181935	27280038	38	98104	APP	*APP*
